# Designed synthesis of double-stage two-dimensional covalent organic frameworks

**DOI:** 10.1038/srep14650

**Published:** 2015-10-12

**Authors:** Xiong Chen, Matthew Addicoat, Enquan Jin, Hong Xu, Taku Hayashi, Fei Xu, Ning Huang, Stephan Irle, Donglin Jiang

**Affiliations:** 1 Department of Materials Molecular Science, Institute for Molecular Science, National Institutes of Natural Sciences, 5-1 Higashiyama, Myodaiji, Okazaki 444-8787, Japan; 2 WPI-Research Initiative-Institute of Transformative Bio-Molecules and Department of Chemistry, Graduate School of Science, Nagoya University, Furo-cho, Chikusa-ku, Nagoya 464-8602, Japan; 3 Department of Physics and Earth Sciences, Jacobs University Bremen, 28759 Germany

## Abstract

Covalent organic frameworks (COFs) are an emerging class of crystalline porous polymers in which organic building blocks are covalently and topologically linked to form extended crystalline polygon structures, constituting a new platform for designing π-electronic porous materials. However, COFs are currently synthesised by a few chemical reactions, limiting the access to and exploration of new structures and properties. The development of new reaction systems that avoid such limitations to expand structural diversity is highly desired. Here we report that COFs can be synthesised via a double-stage connection that polymerises various different building blocks into crystalline polygon architectures, leading to the development of a new type of COFs with enhanced structural complexity and diversity. We show that the double-stage approach not only controls the sequence of building blocks but also allows fine engineering of pore size and shape. This strategy is widely applicable to different polymerisation systems to yield hexagonal, tetragonal and rhombus COFs with predesigned pores and π-arrays.

Covalent organic frameworks (COFs) are an attractive class of crystalline polymers that allow periodic orderings of covalently linked building blocks into porous frameworks in a topologically defined manner^1^,^2^,^3^,^4^,^5^,^6^. COFs are unique in that they possess ordered π-columns and discrete nanopores; these structural features are not available for common polymers and amorphous porous polymers. Owing to these structural characters, COFs provide a useful platform for designing novel crystalline polymers and exploring new functions. COFs have shown their outstanding properties and functions in gas adsorption^2^,^3^,^4^,^6^,^7^,^8^,^9^,^10^,^11^,^12^,^13^,^14^,^15^,^16^,^17^, catalysis^18^,^19^,^20^,^21^, semiconduction^5^,^22^,^23^,^24^,^25^,^26^,^27^, chemosensing^28^, ion conduction^29^, photoconduction^22^,^23^,^25^,^26^,^30^, photoenergy conversion^31^,^32^,^33^ and electric energy storage^34^,^35^. However, COFs are currently synthesised through only a few chemical reactions, limiting the access to and exploration of new structures and functions.^1^ The linkages developed to date for the synthesis of COFs are classified as boronate^2^,^3^,^4^,^5^,^6^,^22^,^23^,^24^,^25^,^36^,^37^,^38^,^39^,^40^,^41^, boroxine^42^,^43^,^44^, borosilicate^8^, imine^45^,^46^,^47^,^48^,^49^,^50^,^51^,^52^,^53^,^54^, hydrazone^11^,^20^,^55^, squaraine^56^, phenazine^57^, azine^28^, imide bonds^58^. In this context, the development of new strategy that avoids such limitations and allows multiple linkages for the construction of COFs is highly desirable.

Herein we report the development of a double-stage approach that enables the use of two types of linkage for the construction of COFs. We demonstrate that this strategy is widely applicable to different polymerisation systems to produce hexagonal, tetragonal and rhombus COFs with high crystallinity and porosity. The double-stage COFs allow the integration of various building blocks into sequenced super lattices and enable the engineering of π-arrays and pore size and shape. Therefore, this double-stage strategy significantly increases the diversity and complexity of COFs, leading to a new phase in designing COF structures and functions.

## Results

### Molecular design principle

**Figure 1:**
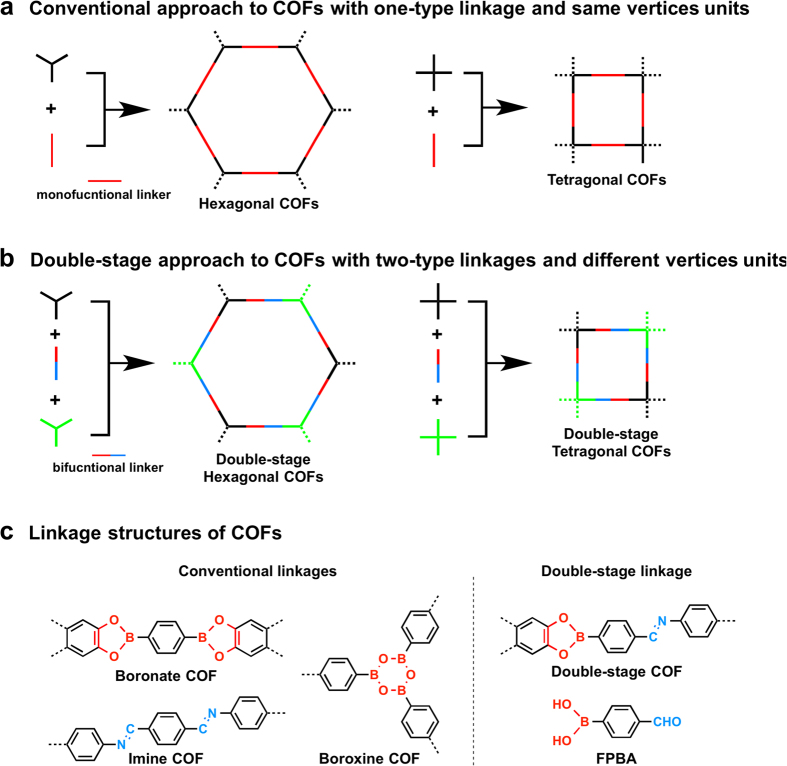
Chemical approach to COFs. (**a**) Schematic representation of the approaches to the conventional hexagonal and tetragonal COFs with monofucntional linkers made of two kinds of monomers. (**b**) Schematic representation of the double-stage approach to COFs with bifucntional linkers made of three different building blocks. (**c**) Typical linkage structures of boronate, boroxine and imine COFs, and linkage structure of the double-stage COF along with the structure of the bifunctional linker 4-formylphenylboronic acid (FPBA).

The key to the double-stage connection strategy is the linker monomer unit that consists of two different reactive groups. This bifunctional linker allows the covalent link of two types of vertices or edge monomers into polygon skeletons. Figure 1a shows the common polymerisation scheme that utilises monofunctional linkers to form lattice structures of COFs, whereas the hexagonal and tetragonal COFs consist of one-type vertices units. By contrast, the double-stage approach uses bifunctional linkers that introduce two different linkages to the architecture and thus enables the sequenced link of two vertices or edge units into the hexagonal or tetragonal skeletons (Fig. 1b). The double-stage COFs thus contain three different components in the lattice. This double-stage lattice structure increases the diversity and complexity of COFs, thus providing a new phase for structural design and functional exploration.

We developed bifunctional linkers with boronic acid and aldehyde functionalities, which reacted with diol and amine units to form boronate and imine linkages, respectively (Fig. 1c). The boronic acid and diol has been developed for the synthesis of boronate-linked COFs, whereas the amine and aldehyde has been used for the preparation of imine-linked COFs. The combination of these boronate and imine linkages using a bifunctional linker unit thus connects two different vertices units with diol and amino functionalities into a single COF material.

We demonstrated this strategy by using a bifunctional linker, i.e., 4-formylphenylboronic acid (Fig. 1c, FPBA), which has one boronic acid and one aldehyde group at the 1,4-positions of a phenyl ring. The boronic acid group can form bronate and boroxine linkages^2^,^3^,^4^,^5^,^6^,^36^,^37^,^38^,^39^,^40^,^41^,^42^,^43^,^44^, whereas the aldehyde unit yields imine^45^,^46^,^47^,^48^,^49^,^50^,^51^,^52^,^53^,^54^ and hydrazone linkages^11^,^20^,^55^. The combination of FPBA and monomers with different reactive units enables the formation of COFs with double-stage connections, including boronate-imine, boronate-hydrazone, and boroxine-imine linkages. Remarkably, this synthetic strategy allows the construction of COFs with different polygon topology, including hexagonal, tetragonal, and rhombus frameworks, leading to the generation of new COF structures with increased complexity.

### Hexagonal COFs

**Figure 2:**
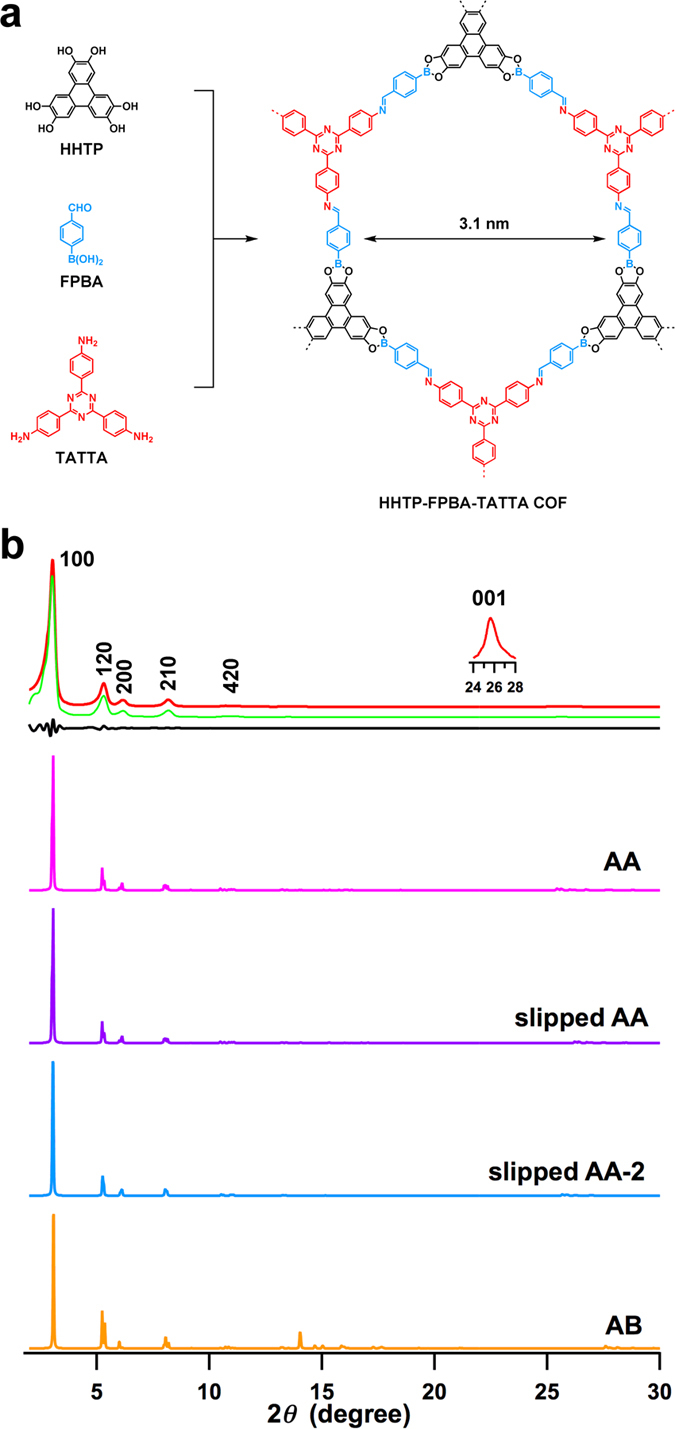
A double-stage hexagonal COF. (**a**) The schematic representation of the synthesis of the hexagonal HHTP-FPBA-TATTA COF. (**b**) XRD patterns of the HHTP-FPBA-TATTA COF (red: experimentally observed, green: Pawley refinement, black: their difference, magenta: simulated with eclipsed AA stacking mode, purple: simulated with slipped AA stacking mode, blue: simulated with slipped AA-2 stacking mode, orange: simulated with staggered AB stacking mode).

We challenged the synthesis of a hexagonal COF, by employing two *C*_3_-symmetric monomers 2,3,6,7,10,11-hexahydroxytriphenylene (HHTP) with diol units and 4,4′,4″-(1,3,5-triazine-2,4,6-triyl)trianiline (TATTA) with amino groups as the vertices for the polymerisation with FPBA (Fig. 2). The FPBA monomer has been employed for the synthesis of hexagonal COFs but with structures that are different from this study^58^. The resulting HHTP-FPBA-TATTA COF assumes a hexagonal structure with mesopore of 3.1 nm in size. In one hexagonal macrocycle, FPBA units (blue) occupy the six edges, whereas three triphenylene (black) units and three triphenyltriazine (red) moieties alternately locate the vertices (Fig. 2a). Compared to conventional COFs that are made of two monomer units, the HHTP-FPBA-TATTA COF consists of three different building blocks, leading to the generation of a new type of hexagonal COFs with enhanced complexity in structure, including the stacked π-columns and channel walls.

We optimised the polymerisation conditions by tuning solvent and reaction time (for all of COFs, see Figures S1–S12). Under optimal conditions, the HHTP-FPBA-TATTA COF was synthesised by heating a mixture of HHTP, TATTA and FPBA (1/1/3 by molar ratio) in dioxane/mesitylene (1/1 by vol.) at 120 °C for 3 days and isolated as a light yellow solid in 89% yield. Infrared spectroscopy (IR) indicates the formation of boronate linkages by showing their vibration bands at 1359 cm^−1^ (B–O), 1243 cm^−1^ (C–O) and 1014 cm^−1^ (B–C), and imine linkages with their characteristic vibration band at 1621 cm^−1^ (Figure S13, Table S1). Elemental analysis results confirmed that the C, H and N contents of the COFs were close to the theoretical values expected for an infinite 2D sheet (Table S2). The HHTP-FPBA-TATTA COF (Fig. 2b, red curve) exhibited prominent X-ray diffraction (XRD) peaks at 3.04°, 5.30°, 6.14°, 8.16°, 10.72° and 25.64°, which were assigned to the (100), (120), (200), (210), (420) and (001) facets, respectively. Pawley refinements (green curve) confirmed the above peak assignments as evidenced by their negligible differences (black curves) (For parameters, see Table S3). The presence of the (001) facet indicates that the 2D COF has periodic order in all three dimensions.

In order to resolve the crystal structure, we utilised density-functional tight-binding (DFTB) method including Lennard–Jones (LJ) dispersion for the optimisation of sheet conformation stacking structure. Table 1 summarises the lattice parameters and total crystal stacking energy per unit cell per layer (For detailed parameters, see Tables S4, S5). For the monolayer, the obtained optimal cell length was *a* = *b* = 33.8 Å (Table S4). In the stacking structure, we optimised four different stacking modes, including eclipsed AA, slipped AA, slipped AA-2 and staggered AB modes, to determine which mode is the most stable one (Table 1, Table S5). The slipped AA-2 mode has a total crystal stacking energy of −90.35 kcal mol^−1^, which is higher than those of eclipsed AA (−88.83 kcal mol^−1^) and slipped AA (−89.84 kcal mol^−1^) modes and much higher than that of staggered AB stacking mode (−36.32 kcal mol^−1^). The slipped AA-2 mode yields an XRD pattern (Fig. 2b, blue curve) that is consistent with the experimentally observed curves, whereas the XRD patterns simulated with the eclipsed AA (magenta curve) and slipped AA stacking modes (purple curve) do not show significant difference from that of slipped AA-2 mode. By contrast, the staggered AB mode (orange curve) cannot reproduce the XRD pattern, in which the peaks at 14°–18° are absent in the experimentally observed XRD profile.

**Figure 3:**
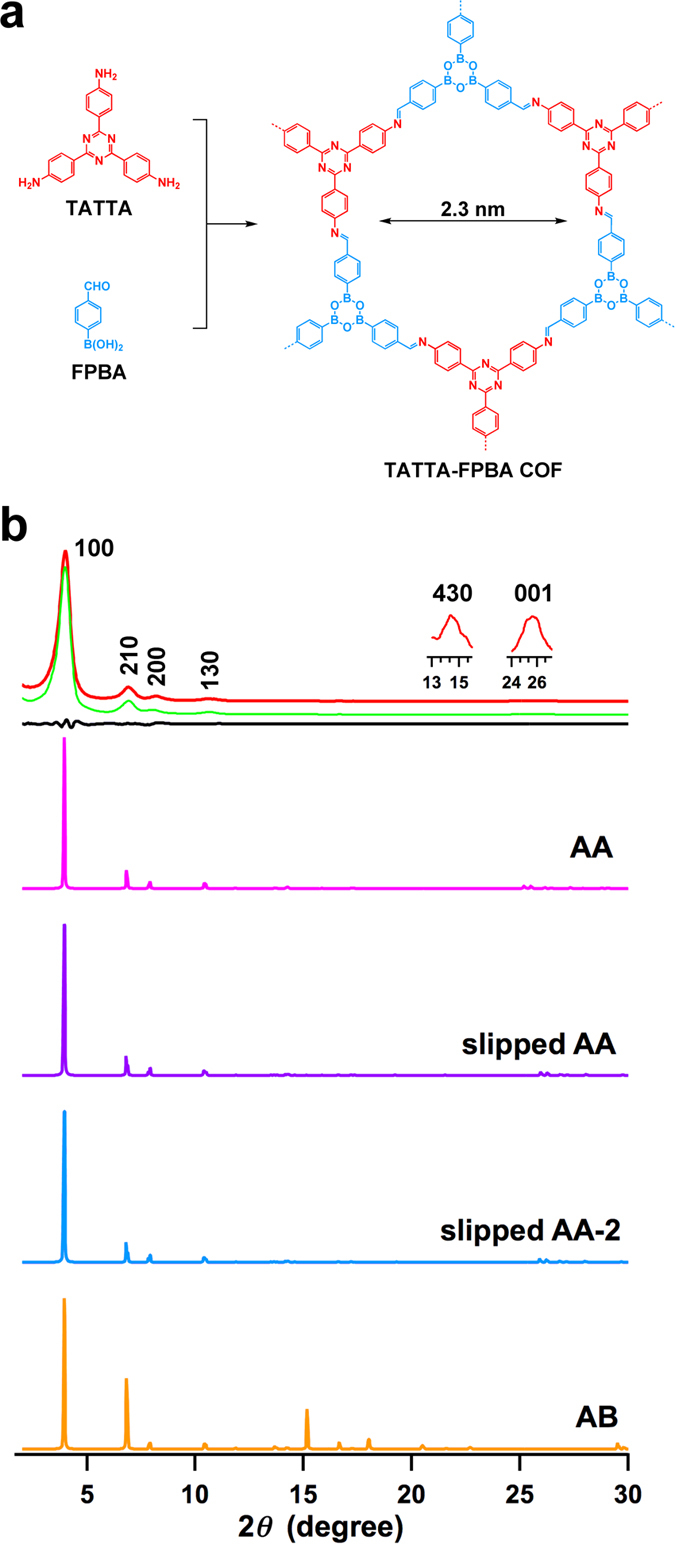
A double-stage self-condensed hexagonal COF. (**a**) The schematic representation of the synthesis of the hexagonal TATTA-FPBA COF. (**b**) XRD patterns of the TATTA-FPBA COF (red: experimentally observed, green: Pawley refinement, black: their difference, magenta: simulated with eclipsed AA stacking mode, purple: simulated with slipped AA stacking mode, blue: simulated with slipped AA-2 stacking mode, orange: simulated with staggered AB stacking mode).

With the above results, we designed the synthesis of another type of hexagonal COF with FPBA and TATTA as building blocks. In this COF, the aldehyde unit of FPBA reacts with TATTA to form imine linkages, whereas the boronic acid unit of FPBA is self-condensed via trimerisation to form boroxine linkages (Fig. 3a). We optimised the polymerisation conditions and synthesised highly crystalline TATTA-FPBA COF in 85% yield as a yellow solid through a three-day reaction of FPBA and TATTA (3/1 by molar ratio) in dioxane/mesitylene (1/9 by vol.) at 120 °C (Figure S2). IR spectroscopy identified the formation of imine linkage with vibration band at 1625 cm^−1^ and boroxine unit with vibration bands at 1363 (B–O), 1016 (B–C) and 713 cm^−1^ (B_3_O_3_) (Table S1, Figure S13).

The TATTA-FPBA COF (Fig. 3b, red curve) exhibited XRD peaks at 4.00°, 6.90°, 8.16°, 10.66°, 14.36° and 25.60°, which were assigned to the (100), (120), (200), (210), (420) and (001) facets, respectively. Pawley refinement (green curve) confirmed the correctness of these peak assignments as evident by a negligible difference (black curve). The DFTB calculations resulted in the monolayer parameters of *a* = *b* = 26.0 Å. Among the stacking modes (Table 1, Table S6), the eclipsed AA stacking mode exhibited a total crystal stacking energy of −63.88 kcal mol^−1^, which is higher than those of slipped AA (−63.73 kcal mol^−1^) and slipped AA-2 (−63.57 kcal mol^−1^) and much higher than that of staggered AB stacking mode (−32.72 kcal mol^−1^). Therefore, the eclipsed AA stacking mode is the most stable structure of the TATTA-FPBA COF. XRD patterns simulated using eclipsed AA (Fig. 3b, magenta curve), slipped AA (purple curve) and slipped AA-2 (blue curve) reproduced the experimentally observed XRD pattern. In contrast, the XRD pattern simulated from the staggered AB stacking mode (orange curve) differs from the experimentally observed XRD profile.

### Rhombus COFs

The above topology design takes advantage of *C*_3_-symmetric vertices and *C*_2_-linkers for the construction of hexagonal COFs. The double-stage strategy is applicable to the design of rhombic COFs by utilising *C*_2_- and *C*_4_-symmetric units as the vertices of the polygon networks. Conventional rhombic COFs have been designed by using *C*_2_-symmetric vertices and *C*_2_-symmetric linker, in which the *C*_2_-symmetric vertices that require four functional groups arranged at an angle of 60° or 120° are limited to only a few organic units. By contrast, the double-stage strategy enables the integration of another *C*_4_-symmetric vertex into the lattice, thus significantly increasing the availability of building blocks and the diversity of rhombic COFs for structural and functional designs.

**Figure 4:**
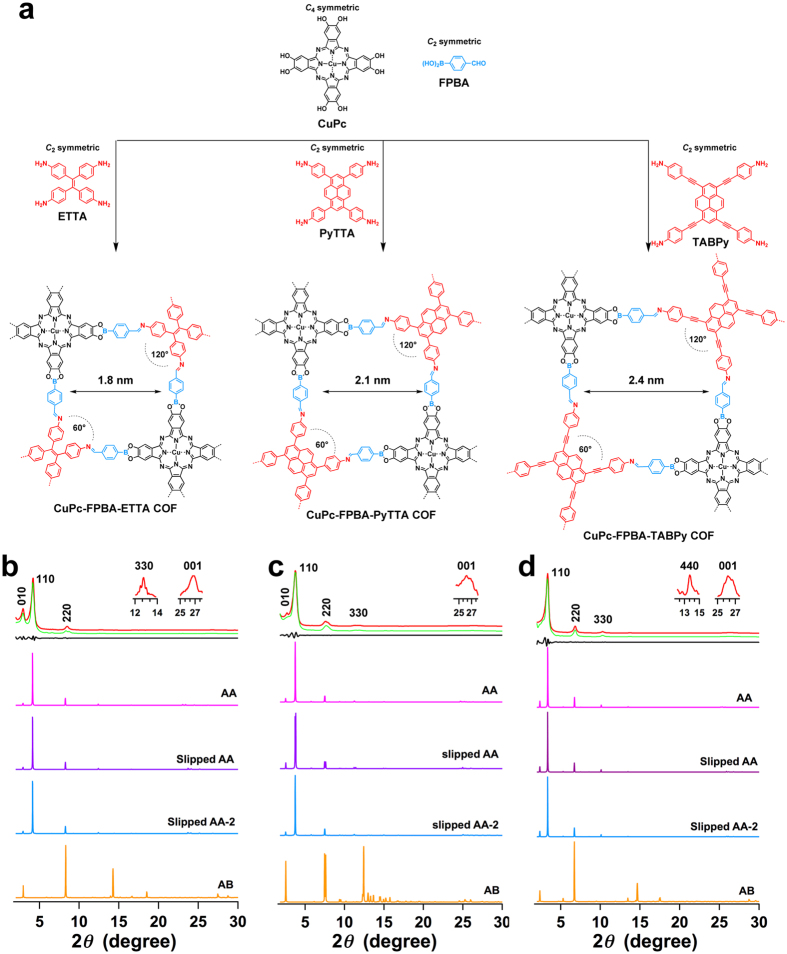
Double-stage rhombic COFs. (**a**) The schematic representations of the synthesis of the rhombic CuPc-FPBA-ETTA COF, CuPc-FPBA-PyTTA COF and CuPc-FPBA-TABPy COF. (**b–d**) XRD patterns of (**b**) CuPc-FPBA-ETTA COF, (**c**) CuPc-FPBA-PyTTA COF and (**d**) CuPc-FPBA-TABPy COF (red: experimentally observed, green: Pawley refinement, black: their difference, magenta: simulated with eclipsed AA stacking mode, purple: simulated with slipped AA stacking mode, blue: simulated with slipped AA-2 stacking mode, orange: simulated with staggered AB stacking mode).

We demonstrated the double-stage rhombic COFs by using a *C*_4_-symmetric phthalocyanine unit with four pairs of diol reactive groups as vertices and tuning another vertices with amino-functionalised *C*_2_-symmetric building blocks, including pyrene and tetraphenylethene derivatives. Figure 4a shows the schematic route to three different rhombic COFs, in which three building blocks are linked covalently into the 2D polygon layers and form the layered frameworks. The polymerisation reactions were conducted by heating the mixture of three components containing (2,3,9,10,16,17,23,24-octahydroxyphthalocyaninato) copper (II) (CuPc), FPBA and 4,4′,4″,4″′-(ethene-1,1,2,2-tetrayl)tetraaniline (ETTA) or 4,4′,4″,4″′-(pyrene-1,3,6,8-tetrayl)tetraaniline (PyTTA) or 1,3,6,8-tetrakis(aminobenzoic)pyrene (TABPy) in DMAc/*o*-DCB (2/1 or 3/1 by vol.) at 120 °C for 7 days. The CuPc-FPBA-ETTA COF, CuPc-FPBA-PyTTA COF and CuPc-FPBA-TABPy COF were isolated as dark green solids in 80%, 91% and 83% yields, respectively (Figures S3–S5). The formation of the double linkages was identified by IR spectra, which showed the new vibration bands of boronate units at 1342 (B–O), 1287 (C–O) and 1087 cm^−1^ (B–C) for the CuPc-FPBA-ETTA COF, 1342 (B–O), 1287 (C–O) and 1086 cm^−1^ (B–C) for the CuPc-FPBA-PyTTA COF, and 1341 (B–O), 1287 (C–O), and 1087 cm^−1^ (B–C) for the CuPc-FPBA-TABPy COF, respectively (Figure S13, Table S1). The new vibration bands assigned to the imine bonds at appeared at 1619, 1618 and 1618 cm^−1^ for the CuPc-FPBA-ETTA COF, CuPc-FPBA-PyTTA COF and CuPc-FPBA-TABPy COF, respectively (Figure S13, Table S1).

The CuPc-FPBA-ETTA COF exhibited XRD peaks at 2.94°, 4.18°, 8.46°, 12.74° and 26.78°, which were assigned to the (010), (110), (220), (330) and (001) facets, respectively (Fig. 4b, red curve). The CuPc-FPBA-PyTTA COF displayed diffractions at 2.66°, 3.78°, 7.64°, 11.30° and 26.24°, which were assigned to the (010), (110), (220), (330) and (001) facets, respectively (Fig. 4c, red curve). The CuPc-FPBA-TABPy COF showed XRD peaks at 3.36°, 6.82°, 10.26°, 13.68° and 26.18°, which were attributed to the (110), (220), (330), (440) and (001) facets, respectively (Fig. 4d, red curve). The presence of the 001 facets indicates that these rhombic COFs are highly ordered not only over the 2D polygon sheets but also along the stacking direction. Pawley refinements (Fig. 4b–d, green curves) reproduced the experimentally observed XRD patterns with negligible difference (black curves), suggesting the correctness of the above peak assignments (Table S3).

As the length of the *C*_2_-symmetric vertices was increased from ETTA to PyTTA and TABPy, the lattice size of the resulting COFs increased. The optimal lattice parameter is *a* = 33.49 Å, *b* = 33.04 Å and *c* = 3.75 Å for the CuPc-FPBA-ETTA COF, *a* = 35.11 Å, *b* = 32.11 Å and *c* = 3.57 Å for the CuPc-FPBA-PyTTA COF and *a* = 37.23 Å, *b* = 37.15 Å and *c* = 3.44 Å for the CuPc-FPBA-TABPy COF (Table 1). For the CuPc-FPBA-ETTA COF (Table S7), the slipped AA-2 stacking mode results in a total crystal stacking energy of −81.79 kcal mol^−1^, which is higher than those of eclipsed AA (−80.24 kcal mol^−1^) and slipped AA (−81.12 kcal mol^−1^) and much higher than that of staggered AB stacking mode (−64.57 kcal mol^−1^). In the cases of the CuPc-FPBA-PyTTA COF (Table S8) and CuPc-FPBA-TABPy COF (Table S9), the slipped AA stacking modes exhibited the crystal stacking energy of −132.46 kcal mol^−1^ and −152.26 kcal mol^−1^, respectively, which are the highest among all the stacking modes, including the eclipsed AA, slipped AA-2 and staggered AB stacking modes (Table 1).

Simulations of the XRD patterns using the slipped AA-2 stacking mode (Fig. 4b, blue curve) for the CuPc-FPBA-ETTA COF and the slipped AA stacking modes (Fig. 4c,d, purple curves) for the CuPc-FPBA-PyTTA COF and CuPc-FPBA-TABPy reproduced the XRD patterns of the CuPc-FPBA-ETTA COF, CuPc-FPBA-PyTTA COF and CuPc-FPBA-TABPy COF, respectively. By contrast, the staggered AB stacking modes (Fig. 4b–d, orange curves) cannot reproduce the XRD patterns. The optimal structures of the double-stage rhombic COFs are formed by maximising the total crystal stacking energy.

### Tetragonal COFs

**Figure 5:**
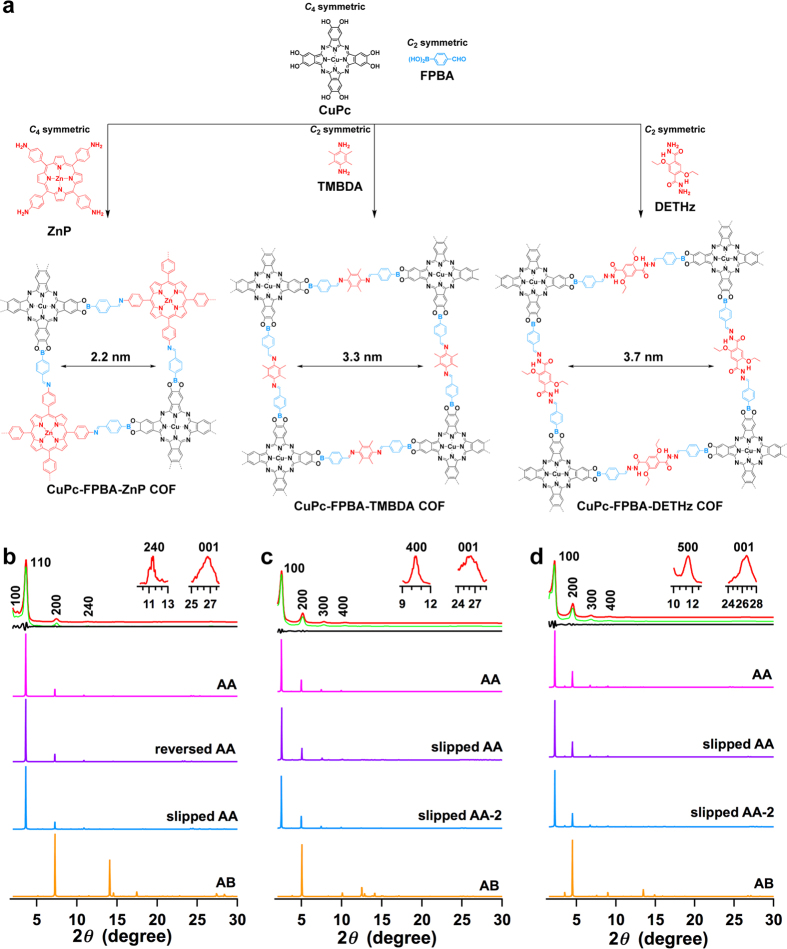
Double-stage tetragonal COFs. (**a**) The schematic representations of the synthesis of the tetragonal CuPc-FPBA-ZnP COF, CuPc-FPBA-TMBDA COF and CuPc-FPBA-DETHz COF. (**b–d**) XRD patterns of (**b**) CuPc-FPBA-ZnP COF, (**c**) CuPc-FPBA-TMBDA COF and (**d**) CuPc-FPBA-DETHz COF (red: experimentally observed, green: Pawley refinement, black: their difference, magenta: simulated with eclipsed or reversed AA stacking mode, purple: simulated with slipped AA stacking mode, blue: simulated with slipped AA-2 stacking mode, orange: simulated with staggered AB stacking mode).

The double-stage strategy is applicable to various organic building blocks with different geometries and functionalities, leading to the generation of various COFs with different π-systems and porous structures. To demonstrate the versatility, we designed the synthesis of tetragonal COFs by employing *C*_4_-symmetric CuPc with diol units as vertices and amino-functionalised building blocks as building blocks for the synthesis of three-type tetragonal COFs (Fig. 5).

Firstly, the *C*_4_-symmetric porphyrin monomer was synthesised as the vertices and the polymerisation with CuPc and FPBA resulted in a tetragonal COF (Fig. 5a, CuPc-FPBA-ZnP COF) with latticed porphyrin and phthalocyanine arrays. The polymerisation was conducted under solvothermal conditions by heating the mixture of zinc 5,10,15,20-tetrakis(4′-tetraphenylamino) porphyrin (ZnP), CuPc and FPBA in DMAc/*o*-DCB (2/1 by vol.) at 120 °C for 7 days. The resulting COF was isolated as a dark green solid in 76% yield (Figure S6). IR spectrum indicates the formation of boronate linkages by showing their vibration band at 1342 (B–O), 1284 (C–O) and 1084 cm^−1^ (B–C) assigned to the boronate unit and bands at 1604 cm^−1^ assigned to the imine unit (Figure S13, Table S1). XRD pattern of the CuPc-FPBA-ZnP COF displays peaks at 2.60°, 3.66°, 7.42°, 11.42° and 26.68°, which are attributed to the (100), (110), (220), (240) and (001) facets, respectively (Fig. 5b, red curve). The Pawley refined profile (green curve) is consistent with the experimentally observed curve, as evidenced by the negligible difference (black curve), indicating the correctness of the above XRD peak assignment. DFTB calculations (Table S10) gave rise to four stacking modes, including eclipsed AA, reversed AA, slipped AA and staggered AB (Table 1). The slipped AA stacking mode gives rise to a total crystal stacking energy of −127.18 kcal mol^−1^, which is higher than those of eclipsed AA (−125.39 kcal mol^−1^), reversed AA (−110.77 kcal mol^−1^) and much higher than that of staggered AB stacking mode (−65.90 kcal mol^−1^). Therefore, among these stacking modes, the slipped AA stacking mode gives rise to the most stable structure. The XRD patterns simulated with slipped AA (blue curve), eclipsed AA (magenta curve) and reversed AA (purple curve) can reproduce the observed curve, whereas the staggered AB mode (orange curve) causes a significant deviation in the XRD pattern.

Secondly, by developing the *C*_2_-symmetric vertices 2,3,5,6-tetramethylbenzene-1,4-diamine (TMBDA) with two amino groups as the building blocks, we observed that the amino-functionalised units could serve as the edges of the double-stage COF (Fig. 5a, CuPc-FPBA-TMBDA COF). By virtue of two units in the edges, the resulting COF significantly expands the lattice size. For example, the CuPc-FPBA-TMBDA COF has a larger pore size of 3.3 nm. The CuPc-FPBA-TMBDA COF was synthesised by condensation of CuPc, TMBDA and FPBA in DMAc/*o*-DCB (2/1 by vol.) at 120 °C for 7 days and was isolated as a dark green solid in 84% yield (Figure S7). The formation of the double linkages was identified by IR spectra, which showed the vibration bands at 1340 (B–O), 1287 (C–O) and 1086 (B–C) assigned to the boronate unit and bands at 1633 cm^−1^ assigned to the imine unit (Figure S13, Table S1). The CuPc-FPBA-TMBDA COF exhibited XRD peaks at 2.50°, 5.14°, 7.80°, 10.42° and 26.14°, which can be assigned to the (100), (200), (300), (400) and (001) facets, respectively (Fig. 5c, red curve). DFTB calculations (Table S11) revealed that the slipped AA stacking mode was the most stable form; it has a total crystal stacking energy of −116.73 kcal mol^−1^, which is higher than those of eclipsed AA (−110.41 kcal mol^−1^), slipped AA-2 (−114.23 kcal mol^−1^) and much higher than that of staggered AB stacking mode (−41.27 kcal mol^−1^). XRD patterns simulated using the slipped AA (blue curve) and eclipsed AA stacking modes (magenta curve) reproduced the experimentally observed curve, whereas the simulation of XRD profile (orange curve) using the staggered AB mode caused significant deviation from the experimentally observed curve.

Finally, we designed another type of double-stage COF by using hydrazone linkage to replace imine unit (Fig. 4a, CuPc-FPBA-DETHz COF), in which the hydrazone units locate at the edges of the polygon framework. The CuPc-FPBA-DETHz COF was synthesised through the reaction of 2,5-diethoxyterephthalohydrazide (DETHz), CuPc and FFBA in DMAc/*o*-DCB (1/3 by vol.) at 120 °C for 7 days and was isolated as a dark green solid in 84% yield (Figure S8). IR spectroscopy revealed the formation of boronate linkages by showing their vibration band at 1342 (B–O), 1289 (C–O) and 1087 cm^−1^ (B–C) assigned to the boronate unit and bands at 1609 cm^−1^ assigned to the imine unit (Figure S13, Table 1). The CuPc-FPBA-DETHz COF displayed XRD diffractions at 2.22°, 4.52°, 6.88°, 9.20°, 11.54° and 26.60°, which were assigned to the (100), (200), (300), (400), (500) and (001) facets, respectively (Fig. 5d, red curve). The Pawley refinement (green curve) reproduced the XRD pattern well with very small difference (black curves). DFTB calculations (Table S11) indicated that the slipped AA stacking mode was the most stable form, which has a total crystal stacking energy of −134.48 kcal mol^−1^. This value is higher than those of eclipsed AA (−104.95 kcal mol^−1^) and slipped AA-2 (−128.85 kcal mol^−1^), and much higher than that of staggered AB stacking mode (−59.72 kcal mol^−1^). XRD pattern simulated using the slipped AA (blue curve) reproduced the experimentally observed curve, whereas the simulation using the staggered AB mode (orange curve) leads to a significant deviation from the experimentally observed curve.

The CuPc-FPBA-DETHz COF consists of long hydrazone unit at the edges of the polygons, thus having a large pore size of 3.7 nm. Notably, this pore size is the largest among the phthalocyanine COFs reported to date^1^.

### Tuning of bifunctional linkers

**Figure 6:**
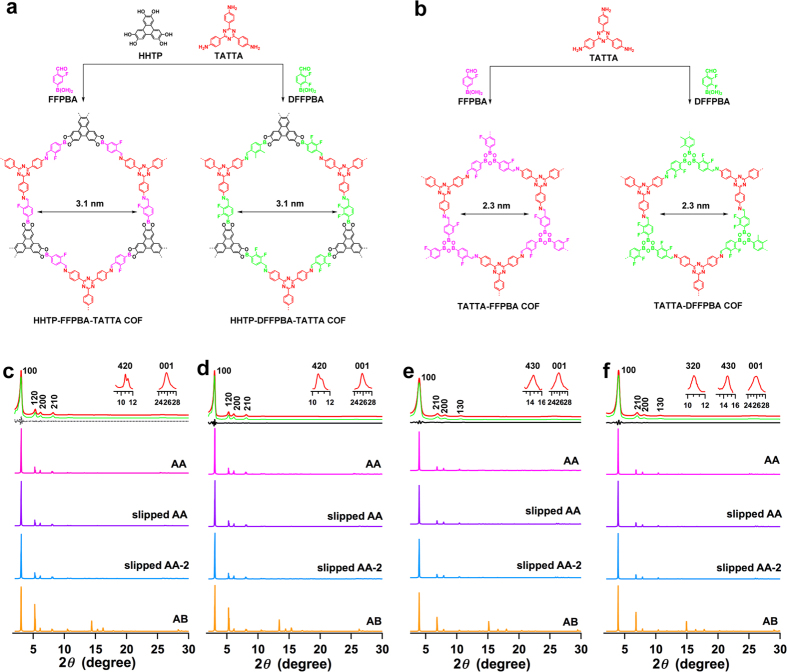
Double-stage hexagonal COFs with fluorinated linkers. (**a**,**b**) The schematic representations of (**a**) the synthesis of the HHTP-FFPBA-TATTA COF, HHTP-DFFPBA-TATTA COF and (**b**) the synthesis of the TATTA-FFPBA COF, TATTA-DFFPBA COF. (**c–f**) XRD patterns of (**c**) HHTP-FFPBA-TATTA COF, (**d**) HHTP-DFFPBA-TATTA COF, (**e**) TATTA-FFPBA COF and (**f**) TATTA-DFFPBA COF (red: experimentally observed, green: Pawley refinement, black: their difference, magenta: simulated with eclipsed AA stacking mode, purple: simulated with slipped AA stacking mode, blue: simulated with slipped AA-2 stacking mode, orange: simulated with staggered AB stacking mode).

We observed that the bifunctional linker FPBA can be further tuned. For this purpose, we developed fluoro-substituted (3-fluoro-4-formylphenyl)boronic acid (FFPBA) and (2,3-difluoro-4-formylphenyl)boronic acid (DFFPBA) as the bifunctional linker for the synthesis of double-stage COFs (Fig. 6).

We demonstrated the strategy by replacing FPBA with FFPBA and DFFPBA for the synthesis of hexagonal COFs. The HHTP-FFPBA-TATTA COF and HHTP-DFFPBA-TATTA COF (Fig. 6a) were prepared as light yellow solids in 85% and 81% yields, respectively, by condensation of HHTP, TATTA, and FFPBA or DFFPBA under solvothermal conditions in dioxane/mesitylene (1/3 by vol.) at 120 °C for 7 days (Figures S9, S10). By contrast, the TATTA-FFPBA COF and TATTA-DFFPBA COF (Fig. 6b) were prepared in 83% and 80% yields, respectively, through the condensation of TATTA with FFPBA or DFFPBA under solvothermal conditions in dioxane/mesitylene (1/9 by vol.) at 120 °C for 7 days (Figures S11, S12).

The HHTP-FFPBA-TATTA COF (Fig. 6c, red curve) exhibited XRD peaks at 3.04°, 5.32°, 6.16°, 8.18°, 10.72° and 25.74°, which can be assigned to the (100), (120), (200), (210), (420) and (001) facets, respectively. The presence of (001) facets indicates that The HHTP-FFPBA-TATTA COF has periodic order in all three dimensions. Pawley refined XRD pattern (green curve) indicates that the assignment of the XRD peaks is correct. DFTB calculations (Table S13) revealed that the slipped AA-2 stacking mode was the most stable form, which has a total crystal stacking energy of −90.35 kcal mol^−1^. This stacking energy is higher than those of eclipsed AA (−88.83 kcal mol^−1^), slipped AA (−89.84 kcal mol^−1^) and much higher than that of staggered AB stacking mode (−36.32 kcal kcal mol^−1^). XRD patterns simulated using the slipped AA-2 (Fig. 6c, blue curve), slipped AA (purple curve) and eclipsed AA stacking modes (magenta curve) reproduced the experimentally observed curve, whereas the simulation of XRD profile (orange curve) using the staggered AB mode cannot reproduce the experimentally observed curve.

The HHTP-DFFPBA-TATTA COF (Fig. 6d, red curve) exhibited a similar XRD pattern as that of the HHTP-FPBA-TATTA, by showing peaks at 3.00°, 5.26°, 6.10°, 8.10°, 10.64° and 25.76°, which can be assigned to the (100), (120), (200), (210), (420) and (001) facets, respectively. DFTB calculations (Table S14) suggested that the slipped AA-2 stacking mode was the most stable form; it has a total crystal stacking energy of−90.61 kcal mol^−1^, which is higher than those of eclipsed AA (−89.40 kcal mol^−1^), slipped AA (−89.86 kcal mol^−1^) and much higher than that of staggered AB stacking mode (−39.09 kcal mol^−1^). XRD patterns simulated using the slipped AA-2 (Fig. 6d, blue curve), slipped AA (purple curve) and eclipsed AA stacking modes (magenta curve) reproduced the experimentally observed curve. By contrast, the simulation of XRD profile (orange curve) using the staggered AB mode cannot reproduce the experimentally observed curve.

The TATTA-FFPBA COF (Fig. 6e, red curve) exhibited XRD peaks at 3.94°, 6.92°, 8.08°, 10.62°, 14.56° and 25.78°, which were assigned to the (100), (210), (200), (130), (430) and (001) facets, respectively. Pawley refinement (green curve) confirmed the correctness of these peak assignments as evident by a negligible difference (black curve). Among the stacking modes (Table 1, Table S15), the eclipsed AA stacking mode is the most stable structure of the TATTA-FFPBA COF. The eclipsed AA stacking mode exhibited a total crystal stacking energy of −64.73 kcal mol^−1^, which is higher than those of slipped AA (−64.43 kcal mol^−1^) and slipped AA-2 (−64.47 kcal mol^−1^) and much higher than that of staggered AB stacking mode (−32.84 kcal mol^−1^). XRD patterns simulated using the eclipsed AA (Fig. 6e, magenta curve), slipped AA (purple curve) and slipped AA-2 (blue curve) reproduced the experimentally observed XRD pattern. In contrast, the XRD pattern simulated from the staggered AB stacking mode (orange curve) significantly deviated from the experimentally observed XRD profile.

The TATTA-DFFPBA COF (Fig. 6f, red curve) exhibited XRD peaks at 4.00°, 7.00°, 8.12°, 10.70°, 14.62° and 25.94°, which were assigned to the (100), (210), (200), (130), (430) and (001) facets, respectively. Pawley refinement (green curve) confirmed the correctness of these peak assignments as evident by a negligible difference (black curve). The eclipsed AA stacking mode (Table 1, Table S16) exhibited a total crystal stacking energy of −67.47 kcal mol^−1^, which is higher than those of slipped AA (−67.02 kcal mol^−1^) and slipped AA-2 (−67.06 kcal mol^−1^) and much higher than that of staggered AB stacking mode (−35.40 kcal mol^−1^). This observation suggests that the eclipsed AA stacking mode is the most stable structure of the TATTA-DFFPBA COF. XRD patterns simulated using eclipsed AA (Fig. 6f, magenta curve), slipped AA (purple curve) and slipped AA-2 (blue curve) reproduced the experimentally observed XRD pattern. In contrast, the XRD pattern simulated from the staggered AB stacking mode (orange curve) cannot reproduce the experimentally observed XRD profile.

Based on the above results, we conclude that the double-stage strategy enables the molecular design of COFs with different vertices, edges and linkers. This design flexibility together with the diversity of organic units significantly enhances the structural diversity and complexity of the COF materials.

## Discussions

**Figure 7:**
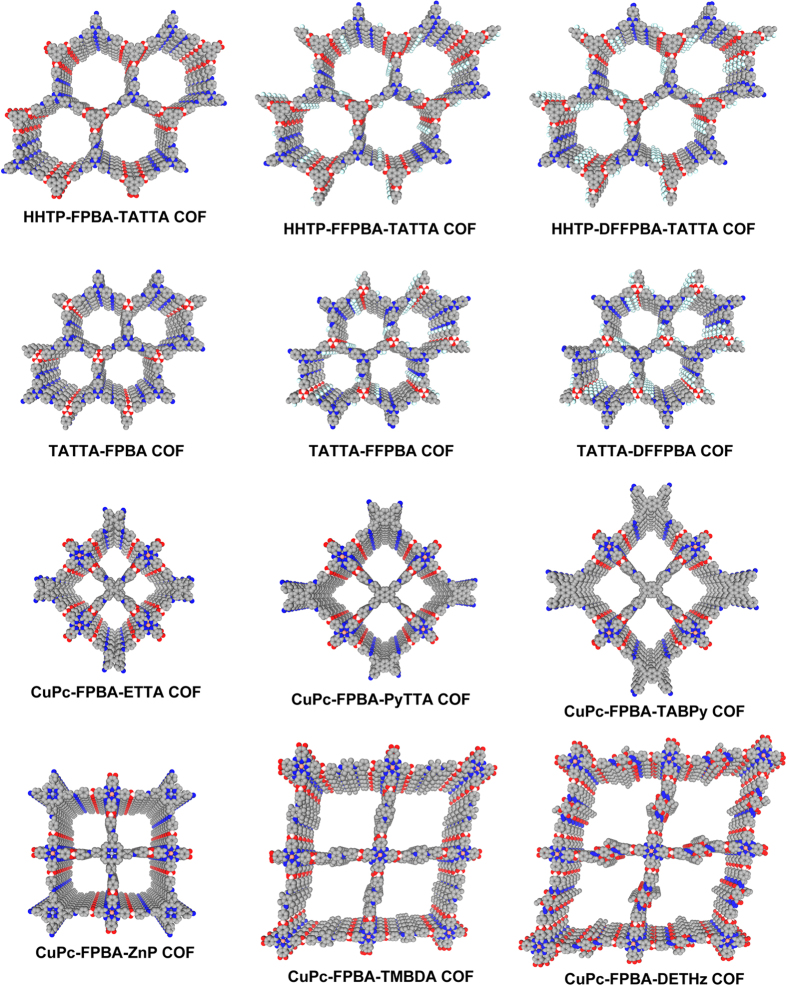
Crystal stacking structure. The 2 × 2 pore structures of the double-stage COFs are presented. The double-stage strategy generates hexagonal, rhombic and tetragonal COFs with different π-column orderings and different pore sizes and shapes, increasing the diversity and complexity of COF structures.

Depending on the geometry of building blocks and the numbers of functional groups, the lattice structures of the COFs can be designed with multiple components; the utilisation of the bifunctional linker increases the number of building blocks in the frameworks that are crucial to fulfill the structural and functional diversity and complexity. For example, the π-columns are increased from two to three types; such increased π-column types would enable the elaborate design of complex systems for high-rate cascade energy transfer or energy conversion. On the other hand, the 1D channels with fine structures and functionalities are useful for the design of tailored interface that is essential to high-performance molecular adsorption, separation and catalysis. In this sense, the double-stage strategy brings the chemistry of COFs to a new phase in the design and exploration of new structures and functions. Figure 7 illustrates the 2 × 2 pores of the stacking structure of the double-stage COFs.

### Crystal stacking energy

In this study, we have developed various π-units for the synthesis of the double-stage 2D COFs. Among them, phthalocyanine, porphyrin, triphenylene, pyrene, tetraphenylethene and triphenyltriazine are typical π-units that are involved in the present strategy for the synthesis of double-stage COFs. Notably, these π-units have been widely utilised in various applications.

We observed that the total crystal stacking energy is highly dependent on the π-units of the COFs (Table 1). A general tendency is that the crystal stacking energy decreases in the order of phthalocyanine > triphenylene > triphenyltriazine. For example, most of CuPc-based COFs have the stacking energy between −116.73 and −152.26 kcal mol^−1^, whereas the crystal stacking energy of the triphenylene-based COFs was approximately –90 kcal mol^−1^ and the triphenyltriazine-based COFs had the crystal stacking energy of approximately –65 kcal mol^−1^. In the same series, the crystal stacking energy is further tuned through the perturbation of other units. Especially for the rhombic COFs, the perturbation with another vertices unit clearly changed the crystal stacking energy. When the vertices unit was changed from ETTA to PyTTA and TABPy, the crystal stacking energy changed from −81.79 to −132.46 and −152.26 kcal mol^−1^.

We also observed the total crystal stacking energy is dependent on the stacking modes. The eclipsed AA, slipped AA and slipped AA-2 stacking modes are stable structures of COFs, whereas the slight difference in energy between these modes is caused by the perturbation of slipped distance between layers. Nevertheless, the perturbation in the slipped distance does not cause a clear change in the XRD peak positions and intensities. This is the reason why these AA stacking modes usually lead to similar XRD patterns. By contrast, the staggered AB stacking mode gives rise to a dramatically decreased stacking energy, as a result of loss of effective stacking between π-units.

### Kinetic studies

The successful synthesis of double-stage linked COFs provides a new platform for the generation of crystalline structures of extended 2D organic polymers. To answer the question how the structure is developed, we evaluated the reaction rate constants under different reaction concentrations and temperatures and chose the HHTP-FPBA-TATTA COF and TATTA-FPBA COF as typical examples^58^,^59^,^60^. For both COFs, we prepared the solutions of monomers at different concentrations and monitored the time-dependent absorbance at 1310 nm, at which both monomer and polymer do not possess inherent absorption bands. Thus, the change of the absorbance at 1310 nm reflects the generation of COF particles that have low solubility and cause the decrease of transparency of the solutions.

In the case of the HHTP-FPBA-TATTA COF at 90 °C, at each concentration, the absorbance at 1310 nm increased as the reaction time was extended (Figure S14a); and the degree of enhancement of absorbance increased as the concentration was increased. From these changes, we evaluated the concentration-dependent initial reaction rate (Figure S14c); the reaction rate was highly dependent on the concentration and exhibited the third power correlation with the concentration. We further investigated the temperature-dependent absorbance changes (Figure S14b). The absorbance at 1310 nm increased as the reaction temperature was increased. We evaluated the activation energy for the polymerisation to synthesize HHTP-FPBA-TATTA COF to be 39.2 kcal mol^−1^ at average (31.5–47.0 kcal mol^−1^), from the pseudo-Arrhenius plot shown in Figure S14d.

In the case of the TATTA-FPBA COF, we conducted similar concentration and temperature-dependent experiments to those of the HHTP-FPBA-TATTA COF. Figure S15a and S15b show the concentration and temperature-dependent absorbance changes, respectively. From these changes, we evaluated the concentration-dependent initial reaction rate (Figure S15c); the reaction rate was in the sixth-power correlation with the concentration. The activation energy for the reaction to prepare the TATTA-FPBA COF was evaluated to be 29.3 kcal mol^−1^ at average (27.3–31.4 kcal mol^−1^) as evaluated form the pseudo-Arrhenius plot (Figure S15d). This low activation energy likely originates from the quick self-condensation of boronic acid groups in the reaction system.

### Band gap engineering

The integration of π-units into the COFs is intriguing for band gap engineering. We estimated the optical band gap using the electronic adsorption spectra of COFs (Figure S16). We observed that the band gap of the COFs is highly dependent on the π-units (Table 2). The phthalocyanine unit is a large π-macrocyle and is effective for generating low band gap COFs. For example, the CuPc-FPBA-ZnP COF, CuPc-FPBA-TMBDA COF and CuPc-FPBA-DETHz COF exhibited band gap as low as 1.360, 1.289 and 1.360 eV, respectively. Similarly, the band gap of the CuPc-FPBA-ETTA COF, CuPc-FPBA-PyTTA COF and CuPc-FPBA-TABPy COF was as low as 1.379, 1.419 and 1.355 eV, respectively. The triphenylene vertices give rise to medium band gap COFs. For example, the HHTP-FPBA-TATTA COF, HHTP-FFPBA-TATTA COF and HHTP-DFFPBA-TATTA COF have band gaps of 1.994, 1.962 and 1.984 eV, respectively. By contrast, the less π-conjugated triphenyltriazine units lead to high band gaps. For example, the TATTA-FPBA COF, TATTA-FFPBA COF and TATTA-DFFPBA COF exhibited band gap as high as 2.362, 2.318 and 2.340 eV, respectively.

To provide further understanding of the highest occupied molecular orbital (HOMO) and the lowest unoccupied molecular orbital (LUMO) levels and the structural factors that control the redox activity of the COFs, we visualised their molecular orbitals. Figures S17–20 illustrate the mapping of HOMO and LUMO for the most stable stacking modes of the COFs. For the phthalocyanine-based COFs, the rhombic CuPc-FPBA-ETTA COF, CuPc-FPBA-PyTTA COF and CuPc-FPBA-TABPy COF exhibited HOMO locating on the ETTA, PyTTA and TABPy units and LUMO lying on the CuPc units (Figure S18a–c). Thus, in the rhombic series, the band gap and redox activity are determined by the two vertices units of the frameworks, whereas the edge units do not involve in the redox diagram. For the tetragonal phthalocyanine-based COFs (Figure S19), the HOMO and LUMO of the CuPc-FPBA-ZnP COF and CuPc-FPBA-DETHz COF locate on the CuPc vertices units (Figure S19a,c). By contrast, the CuPc-FPBA-TMBDA COF has HOMO and LUMO located on the edge unit and CuPc unit, respectively (Figure S19b). The integration of TMBDA unit to the edge part of the polygon introduces two imine linkages that cause the shift of the HOMO level from CuPc to the edge units. In the hexagonal COFs, the HOMO locates on the TATTA units and the LUMO lies on the edge units (Figure S20); this is the same case for all the six hexagonal COFs, including the COFs with fluoro- and difluoro-substituted FPBA linkers (Figure S20). Therefore, in the hexagonal COFs, the redox activity is controlled by the TATTA and edge units. These insights into the energy diagram offer a mechanistic guidance for designing double-stage COFs for optoelectronic applications.

### Porosity and gas adsorption

**Figure 8:**
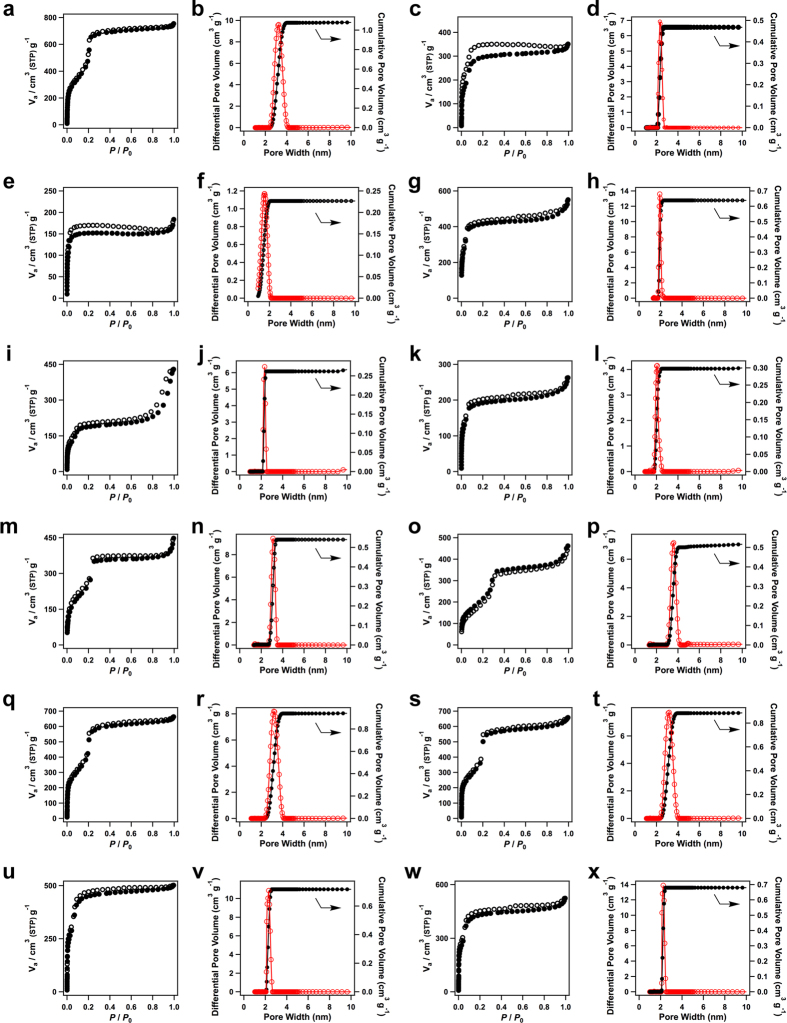
Gas adsorption. (**a**–**x**) Nitrogen sorption curves of (**a**) HHTP-FPBA-TATTA COF, (**c**) TATTA-FPBA COF, (**e**) CuPc-FPBA-ETTA COF, (**g**) CuPc-FPBA-PyTTA COF, (**i**) CuPc-FPBA-TABPy COF, (**k**) CuPc-FPBA-ZnP COF, (**m**) CuPc-FPBA-TMBDA COF, (**o**) CuPc-FPBA-DETHz COF, (**q**) HHTP-FFPBA-TATTA COF, (**s**) HHTP-DFFPBA-TATTA COF, (**u**) TATTA-FFPBA COF, (**w**) TATTA-DFFPBA COF (filled circles: adsorption, open circles: desorption) and pore size and pore size distribution profiles of (**b**) HHTP-FPBA-TATTA COF, (**d**) TATTA-FPBA COF, (**f**) CuPc-FPBA-ETTA COF, (**h**) CuPc-FPBA-PyTTA COF, (**j**) CuPc-FPBA-TABPy COF, (**l**) CuPc-FPBA-ZnP COF, (**n**) CuPc-FPBA-TMBDA COF, (**p**) CuPc-FPBA-DETHz COF, (**r**) HHTP-FFPBA-TATTA COF, (**t**) HHTP-DFFPBA-TATTA COF, (**v**) TATTA-FFPBA COF, (**x**) TATTA-DFFPBA COF (red curves: pore size, black curves: pore size distribution).

The double-stage COFs not only integrate organic units into periodic columnar arrays but also constitute ordered 1D nanosized open channels. These nanochannels are inherent to the COFs, as a result of topology-directed polymerisation and crystallisation of 2D layers into porous frameworks. The open channels of COFs provide nanosized room to accommodate gas and other molecules. To investigate the porosity, nitrogen sorption isotherms were measurements at 77 K. Figure 8 shows the nitrogen sorption isotherm curves of the double-stage COFs and their pore size distribution profiles. Table 3 summarises the surface area, pore size and pore volume. A general tendency is that these double-stage COFs are highly porous materials with large surface areas. Another distinct feature is that these COFs consist of only one type of pores in their frameworks, as evident by their pore size distribution profiles (Fig. 8). The combination of building block makes the pore size tunable ranging from supermicropores to mesopores.

The hexagonal HHTP-FPBA-TATTA COF (Fig. 8a) and TATTA-FPBA COF (Fig. 8c) exhibited type IV sorption curves, which are characteristic of mesoporous materials. The Brunauer–Emmett–Teller (BET) surface area and pore volume for the HHTP-FPBA-TATTA COF were calculated to be 1975 m^2^ g^−1^ and 1.17 cm^3^ g^−1^, respectively. The TATTA-FPBA COF exhibited a BET surface area and pore volume of 1015 m^2^ g^−1^ and 0.54 cm^3^ g^−1^, respectively. Based on the nonlocal density functional theory (NLDFT) calculation from the sorption curves, the pore size of HHTP-FPBA-TATTA COF (Fig. 8b) and TATTA-FPBA COF (Fig. 8d) was estimated to be 3.2 and 2.3 nm, respectively.

Similar to the above hexagonal COFs, COFs with the fluoro- and difluoro-substituted FPBA linkers, including the HHTP-FFPBA-TATTA COF (Fig. 8q), HHTP-DFFPBA-TATTA COF (Fig. 8s), TATTA-FFPBA COF (Fig. 8u) and TATTA-DFFPBA COF (Fig. 8w), were mesoporous polymers and exhibited type IV nitrogen sorption isotherm curves. Their BET surface areas were evaluated to be 1748, 1697, 1582 and 1489 m^2^ g^−1^, whereas the pore volumes were 1.03, 1.02, 0.78 and 0.81 cm^3^ g^−1^, respectively. The pore size distribution profiles suggest that the HHTP-FFPBA-TATTA COF (Fig. 8r), HHTP-DFFPBA-TATTA COF (Fig. 8t), TATTA-FFPBA COF (Fig. 8v) and TATTA-DFFPBA COF (Fig. 8x) have pores size of 3.2, 3.2, 2.3 and 2.3 nm, respectively. These values are close to the theoretical pore sizes.

The rhombic CuPc-FPBA-PyTTA COF (Fig. 8g) and CuPc-FPBA-TABPy COF (Fig. 8i) exhibited type IV sorption isotherm profiles, from which their BET surface areas were evaluated to be 1541 and 667 m^2^ g^−1^, respectively. By using NLDFT method, their pore volumes were estimated to be 0.85 and 0.67 cm^3^ g^−1^, respectively. The CuPc-FPBA-PyTTA COF has a pore size of 2.0 nm (Fig. 8h), whereas the CuPc-FPBA-TABPy COF (Fig. 8j) has a pore size of 2.2 nm. By contrast, the CuPc-FPBA-ETTA COF with small sized vertices is microporous polymer with a large adsorption at low pressure (Fig. 8e). The BET surface area and pore volume were evaluated to be 557 m^2^ g^−1^ and 0.28 cm^3^ g^−1^, respectively. The NLDFT calculations revealed that the CuPc-FPBA-ETTA COF has only one type of micropore with size of 1.5 nm (Fig. 8f).

The tetragonal COFs, including the CuPc-FPBA-ZnP COF (Fig. 8k), CuPc-FPBA-TMBDA COF (Fig. 8m) and CuPc-FPBA-DETHz COF (Fig. 8o), are mesoporous polymers. Their BET surface areas are as high as 642, 1141 and 738 m^2^ g^−1^, respectively. The pore volume and pore size were evaluated to be 0.41, 0.70 and 0.72 cm^3^ g^−1^ and 2.0, 3.1 and 3.6 nm for the CuPc-FPBA-ZnP COF, CuPc-FPBA-TMBDA COF and CuPc-FPBA-DETHz COF, respectively. Notably, the CuPc-FPBA-DETHz COF has the largest pore among the phthalocyanine COFs reported to date.

The above results of surface area, pore size and pore volume indicate that the double-stage COFs allow for elaborate tuning of the pore size and porosity through the design of *C*_2_-, *C*_3_- and *C*_4_-symmetric organic building blocks. The presence of the multiple components in the lattice also introduces chemical complexity to the channel walls, which is useful in designing functional pores for gas storage, molecular separation and catalysis.

## Conclusion

By developing a bifunctional linker, we have successfully designed and synthesised a series of new type of COFs. The double-stage COFs are crystalline porous materials with large surface areas, whereas their π-lattice, pore size and pore shape can be tailored by combining different organic building blocks. Hexagonal, tetragonal and rhombic COFs have been synthesised by using conventional solvothermal conditions in high yields. These double-stage COFs feature enhanced structural complexity regarding to the orderings in π-column arrays and dense functional groups embedded on the pore walls as a result of enhanced numbers of building blocks. We envisage that the double-stage strategy opens a new chemical approach to design COF materials with new structures and functions.

## Methods

### HHTP-FPBA-TATTA COF

A mixture of HHTP, TATTA and FPBA (1/1/3 by molar ratio) in dioxane/mesitylene (1/1 by vol.) in a 10 mL Pyrex tube was degassed through freeze–pump-thaw cycles and sealed under vacuum. The tube was placed in an oven at 120 °C for 7 days. The precipitate was collected by centrifugation, washed with THF and acetone and dried under vacuum to afford HHTP-FPBA-TATTA COF as a light yellow solid in 89% yield.

### TATTA-FPBA COF

A dioxane/mesitylene (1/9 by vol.) mixture of FPBA and TATTA (3/1 by molar ratio) in in a 10 mL Pyrex tube was degassed through freeze–pump-thaw cycles and sealed under vacuum. The tube was placed in an oven at 120 °C for 7 days. The precipitate was collected by centrifugation, washed with THF and acetone and dried under vacuum to afford TATTA-FPBA COF as a light yellow solid in 85% yield.

### CuPc-FPBA-ETTA COF

A mixture of CuPc, FPBA and ETTA (1/4/1 by molar ratio) in a mixture of DMAc/*o*-DCB (2/1 by vol.) in a 10 mL Pyrex tube was degassed through freeze–pump-thaw cycles and sealed under vacuum. The tube was placed in an oven at 120 °C for 7 days. The precipitate was collected by centrifugation, washed with DMAc, THF and acetone, and dried under vacuum to afford CuPc-FPBA-ETTA COF as a dark green solid in 80% yield.

### CuPc-FPBA-PyTTA COF

A mixture of CuPc, FPBA and PyTTA (1/4/1 by molar ratio) in a mixture of DMAc/*o*-DCB (3/1 by vol.) in a 10 mL Pyrex tube was degassed through freeze–pump-thaw cycles and sealed under vacuum. The tube was placed in an oven at 120 °C for 7 days. The precipitate was collected by centrifugation, washed with DMAc, THF, and acetone, and dried under vacuum to afford CuPc-FPBA-PyTTA COF as a dark green solid in 91% yield.

### CuPc-FPBA-TABPy COF

A mixture of CuPc, FPBA and TABPy (1/4/1 by molar ratio) in a mixture of DMAc/*o*-DCB (3/1 by vol.) in a 10 mL Pyrex tube was degassed through freeze–pump-thaw cycles and sealed under vacuum. The tube was placed in an oven at 120 °C for 7 days. The precipitate was collected by centrifugation, washed with DMAc, THF and acetone, and dried under vacuum to afford CuPc-FPBA-TABPy COF as a dark green solid in 83% yield.

### CuPc-FPBA-ZnP COF

A mixture of CuPc, FPBA and ZnP (1/4/1 by molar ratio) in a mixture of DMAc/*o*-DCB (2/1 by vol.) in a 10 mL Pyrex tube was degassed through freeze–pump-thaw cycles and sealed under vacuum. The tube was placed in an oven at 120 °C for 7 days. The precipitate was collected by centrifugation, washed with DMAc, THF and acetone, and dried under vacuum to afford CuPc-FPBA-ZnP COF as a dark green solid in 76% yield.

### CuPc-FPBA-TMBDA COF

A mixture of CuPc, FPBA and TMBDA (1/4/2 by molar ratio) in a mixture of DMAc/*o*-DCB (2/1 by vol.) in a 10 mL Pyrex tube was degassed through freeze–pump-thaw cycles and sealed under vacuum. The tube was placed in an oven at 120 °C for 7 days. The precipitate was collected by centrifugation, washed with DMAc, THF and acetone, and dried under vacuum to afford CuPc-FPBA-TMBDA COF as a dark green solid in 89% yield.

### CuPc-FPBA-DETHz COF

A mixture of CuPc, FPBA and DETHz (1/4/2 by molar ratio) in a mixture of DMAc/*o*-DCB (1/3 by vol.) in a 10 mL Pyrex tube was degassed through freeze–pump-thaw cycles and sealed under vacuum. The tube was placed in an oven at 120 °C for 7 days. The precipitate was collected by centrifugation, washed with DMAc, THF and acetone, and dried under vacuum to afford CuPc-FPBA-DETHz COF as a dark green solid in 84% yield.

### HHTP-FFPBA-TATTA COF

A mixture of HHTP, TATTA and FFPBA (1/1/3 by molar ratio) in a mixture of dioxane/mesitylene (1/3 by vol.) in a 10 mL Pyrex tube was degassed through freeze–pump-thaw cycles and sealed under vacuum. The tube was placed in an oven at 120 °C for 7 days. The precipitate was collected by centrifugation, washed with THF and acetone, and dried under vacuum to afford HHTP-FFPBA-TATTA COF as a light yellow solid in 85% yield.

### HHTP-DFFPBA-TATTA COF

A mixture of HHTP, TATTA and DFFPBA (1/1/3 by molar ratio) in a mixture of dioxane/mesitylene (1/3 by vol.) in a 10 mL Pyrex tube was degassed through freeze–pump-thaw cycles and sealed under vacuum. The tube was placed in an oven at 120 °C for 7 days. The precipitate was collected by centrifugation, washed with THF and acetone, and dried under vacuum to afford HHTP-DFFPBA-TATTA COF as a light yellow solid in 81% yield.

### TATTA-FFPBA COF

A mixture of FFTBA and TATTA (3/1 by molar ratio) in a mixture of dioxane/mesitylene (1/9 by vol.) in a 10 mL Pyrex tube was degassed through freeze–pump-thaw cycles and sealed under vacuum. The tube was placed in an oven at 120 °C for 7 days. The precipitate was collected by centrifugation, washed with THF and acetone, and dried under vacuum to afford TATTA-FFPBA COF as a light yellow solid in 83% yield.

### TATTA-DFFPBA COF

A mixture of DFFTBA and TATTA (3/1 by molar ratio) in a mixture of dioxane/mesitylene (1/9 by vol.) in a 10 mL Pyrex tube was degassed through freeze–pump-thaw cycles and sealed under vacuum. The tube was placed in an oven at 120 °C for 7 days. The precipitate was collected by centrifugation, washed with THF and acetone, and dried under vacuum to afford TATTA-DFFPBA COF as a light yellow solid in 80% yield.

## Additional Information

**How to cite this article**: Chen, X. *et al.* Designed synthesis of double-stage two-dimensional covalent organic frameworks. *Sci. Rep.* **5**, 14650; doi: 10.1038/srep14650 (2015).

## Supplementary Material

Supplementary Information

## Figures and Tables

**Table 1:** Total crystal stacking energy (TCSE) and lattice parameters of double-stage COFs.

COFs	Stacking mode	*a, b*[Å]	*c*[Å]	Slipped distance along*a* and/or *b* directions	TCSE per unit cell perlayer (kcal mol^−1^)
HHTP-FPBA-TATTA	AA	*a* = *b* = 33.73	3.50		−88.83
	slipped AA	*a* = *b* = 33.74	3.40	0.4 Å in *a* and 1.1 Å in *b*	−89.84
	slipped AA-2	*a* = 33.57, *b* = 33.67	3.47	1.0 Å in *b*	−90.35
	AB	*a* = 33.70, *b* = 33.75	3.23		−36.32
TATTA-FPBA	AA	*a* = 25.94, *b* = 25.92	3.54		−63.88
	slipped AA	*a* = 25.97, *b* = 25.99	3.43	0.4 Å in *a* and 1.0 Å in *b*	−63.73
	slipped AA-2	*a* = 25.99, *b* = 25.97	3.44	1.4 Å in *a* and 0.8 Å in *b*	−63.57
	AB	*a* = 25.90, *b* = 25.95	3.03		−32.72
CuPc-FPBA-ETTA	AA	*a* = 33.52, *b* = 33.06	3.86		−80.24
	slipped AA	*a* = 33.51, *b* = 33.03	3.75	1.0 Å in *a*	−81.12
	slipped AA-2	*a* = 33.49, *b* = 33.04	3.75	0.9 Å in *a*	−81.79
	AB	*a* = 33.37, *b* = 29.88	3.25		−64.57
CuPc-FPBA-PyTTA	AA	*a* = 35.10, *b* = 31.99	3.60		−130.74
	slipped AA	*a* = 35.11, *b* = 32.11	3.57	0.7 Å in *a* and 0.4 Å in *b*	−132.46
	slipped AA-2	*a* = 35.06, *b* = 32.12	3.57	0.5 Å in *a* and *b*	−130.71
	AB	*a* = 35.05, *b* = 32.22	3.55		−124.72
CuPc-FPBA-TABPy	AA	*a* = 37.16, *b* = 37.06	3.54		−149.82
	slipped AA	*a* = 37.23, *b* = 37.15	3.44	1.1 Å in *a*	−152.26
	slipped AA-2	*a* = 37.22, *b* = 37.16	3.44	1.1 Å in *b*	−151.96
	AB	*a* = 37.26, *b* = 37.16	3.11		−64.31
CuPc-FPBA-ZnP	AA	*a* = *b* = 34.50	3.67		−125.39
	slipped AA	*a* = *b* = 34.48	3.68	0.1 Å in *a* and *b*	−127.18
	reversed AA	*a* = *b* = 34.53	3.84	CuPc on top of ZnP	−110.77
	AB	*a* = 34.45, *b* = 34.41	3.25		−65.90
CuPc-FPBA-TMBDA	AA	*a* = 35.83, *b* = 35.82	3.72		−110.41
	slipped AA	*a* = 35.79, *b* = 35.75	3.59	0.7 Å in *a* and *b*	−116.73
	slipped AA-2	*a* = 35.86, *b* = 35.91	3.58	0.9 Å in *a* and *b*	−114.23
	AB	*a* = 35.91, *b* = 35.85	3.62		−41.27
CuPc-FPBA-DETHz	AA	*a* = 40.46, *b* = 40.44	3.65		−104.95
	slipped AA	*a* = 40.42, *b* = 40.46	3.33	1.9 Å in *a*	−134.48
	slipped AA-2	*a* = 40.37, *b* = 40.45	3.30	2.8 Å in *a* and 2.4 Å in *b*	−128.85
	AB	*a* = 40.45, *b* = 40.42	3.49		−59.72
HHTP-FFPBA-TATTA	AA	*a* = 33.73, *b* = 33.74	3.50		−88.83
	slipped AA	*a* = *b* = 33.74	3.40	0.4 Å in *a* and 1.0 Å in *b*	−89.84
	slipped AA-2	*a* = 33.70, *b* = 33.72	3.45	0.4 Å in *a* and 1.2 Å in *b*	−90.35
	AB	*a* = *b* = 33.73	3.15		−36.32
HHTP-DFFPBA-TATTA	AA	*a* = b = 33.75	3.50		−89.40
	slipped AA	*a* = 33.68, *b* = 33.67	3.39	0.5 Å in *a* and 1.2 Å in *b*	−89.86
	slipped AA-2	a = *b* = 33.75	3.44	1.3 Å in *a* and 1.1 Å in *b*	−90.61
	AB	*a* = 33.65, *b* = 33.63	3.17		−39.09
TATTA-FFPBA	AA	*a* = 25.99, *b* = 25.97	3.53		−64.73
	slipped AA	*a* = 26.00, *b* = 26.01	3.42	0.4 Å in *a* and 1.0 Å in *b*	−64.43
	slipped AA-2	*a* = 26.07, *b* = 26.02	3.44	1.0 Å in *a* and 0.2 Å in *b*	−64.47
	AB	*a* = 26.00, *b* = 26.02	3.03		−32.84
TATTA-DFFPBA	AA	*a* = 26.00, *b* = 26.03	3.55		−67.47
	slipped AA	*a* = 26.06, *b* = 26.09	3.42	0.4 Å in *a* and 1.0 Å in *b*	−67.02
	slipped AA-2	*a* = 26.05, *b* = 26.06	3.43	1.5 Å in *a* and 1.0 Å in *b*	−67.06
	AB	*a* = 26.04, *b* = 26.01	3.08		−35.40

**Table 2:** Band Gaps of double-stage COFs.

COFs		CalculatedHOMO-LUMOGap (eV)	OpticalBand Gap(eV)
CuPc-based COFs	CuPc-FPBA-ZnP	0.125	1.360
	CuPc-FPBA-TMBDA	1.119	1.289
	CuPc-FPBA-DETHz	1.299	1.360
	CuPc-FPBA-ETTA	0.045	1.379
	CuPc-FPBA-PyTTA	0.822	1.419
	CuPc-FPBA-TABPy	0.100	1.355
Triphenylene-based COFs	HHTP-FPBA-TATTA	2.303	1.994
	HHTP-FFPBA-TATTA	2.190	1.962
	HHTP-DFFPBA-TATTA	2.265	1.984
triphenyltriazine-based COFs	TATTA-FPBA	2.345	2.362
	TATTA-FFPBA	2.070	2.318
	TATTA-DFFPBA	2.147	2.340

**Table 3:** Porosity of double-stage COFs.

COFs		Surface area (m^2^ g^−1^)	Pore size(nm)	Total pore volume(cm^3^ g^−1^)
Hexagonal	HHTP-FPBA-TATTA	1975	3.24	1.17
	TATTA-FPBA	1015	2.34	0.54
Rhombic	CuPc-FPBA-ETTA	557	1.53	0.28
	CuPc-FPBA-PyTTA	1541	2.03	0.85
	CuPc-FPBA-TABPy	667	2.21	0.67
Tetragonal	CuPc-FPBA-ZnP	642	2.01	0.41
	CuPc-FPBA-TMBDA	1141	3.11	0.70
	CuPc-FPBA-DETHz	738	3.55	0.72
Hexagonal	HHTP-FFPBA-TATTA	1748	3.17	1.03
	HHTP-DFFPBA-TATTA	1697	3.17	1.02
	TATTA-FFPBA	1582	2.32	0.78
	TATTA-DFFPBA	1489	2.23	0.81
