# Supplementary material for: Designed synthesis of double-stage two-dimensional covalent organic frameworks
Source: Sci Rep. 2015 Oct 12;5:14650. doi: 10.1038/srep14650 (PMC4600973; doi:10.1038/srep14650)
Supplement: Supplementary Information [file srep14650-s1.pdf]

Supplementary Information

Designed synthesis of double-stage two-dimensional covalent organic frameworks

Xiong Chen[1], Matthew Addicoat[2,3], Enquan Jin[1], Hong Xu[1], Taku hayashi[2], Fei Xu[1], Ning Huang[1], Stephan Irle[2] & Donglin Jiang[1]

[1]Department of Materials Molecular Science, Institute for Molecular Science, National Institutes of Natural Sciences, 5-1 Higashiyama, Myodaiji, Okazaki 444-8787, Japan.

[2]WPI-Research Initiative-Institute of Transformative Bio-Molecules and Department of Chemistry, Graduate School of Science, Nagoya University, Furo-cho, Chikusa-ku, Nagoya 464-8602, Japan.

[3]Department of Physics and Earth Sciences, Jacobs University Bremen, 28759 Germany.

Correspondence and requests for materials should be addressed to D.J. (jiang@ims.ac.jp)

Contents

Materials and Methods -- *S*2

Supplementary Tables -- *S*5

Supplementary Figures -- *S*15

Supplementary References -- *S*29

Materials

n-Butanol (*n*-BuOH), anhydrous *N*, *N*-dimethylacetamide (DMAc, 99.0%), *o*-dichlorobenzene (*o*-DCB), anhydrous acetone (99.5%), tetrahydrofuran (THF), mesitylene, dioxane, acetic acid, hydrochloric acid, sulfuric acid and 4-(4,4,5,5-tetramethyl-1,3,2-dioxaborolan-2-yl)aniline were purchased from Wako Chemicals. 1,1,2,2-Tetraphenylethylene (TPE), 4-ethynylaniline, diethyl 2,5-dihydroxyterephthalate, hydrobromic acid, (3-fluoro-4-formylphenyl)boronic acid (FFPBA) and (2,3-difluoro-4-formylphenyl)boronic acid (DFFPBA) were purchased from Aldrich. Fuming nitric acid, pyrrole, propionic acid, pyridine, *p*-nitrobenzaldehyde, 2,3,5,6-tetramethylbenzene-1,4-diamine (TMBDA), tin (II) chloride dihydrate, 1,2-dimethoxybenzene, boron tribromide solution (BBr_3, 1 M in CH_2Cl_2), chlorosulfonic acid, 4-cyanoacetanilide,1,3,6,8-tetrabromopyrene, palladium tetrakis(triphenyl phosphine), [1,1'-bis(diphenylphosphino)ferrocene]palladium(II) dichloride, triphenylphosphine, triethylamine (TEA), iodoethane, hydrazine monohydrate, raney nickel and 4-formylphenylboronic acid (FPBA) were purchased from TCI Chemicals. *N*, *N*-Dimethyl-2-aminoethanol (DMAE), $Zn(OAc)_2{\cdot}H_2O$, iron(III) chloride, potassium carbonate, copper(I) iodide , potassium iodide and $CuCl_2{\cdot}2H_2O$ were purchased from Kanto Chemicals. 4,5-Dibromo-1,2-dimethoxybenzene was purchased from Alfa Aesar. CuCN was purchased from Nacalai Tesque.

(2,3,9,10,16,17,23,24-Octahydroxyphthalocyaninato) copper (II) (CuPc),[S1] 2,3,6,7,10,11-hexahydroxytriphenylene (HHTP),[S2] 4,4',4'',4'''-(ethene-1,1,2,2-tetrayl)tetraaniline (ETTA),[S3] 4,4',4'',4'''-(pyrene-1,3,6,8-tetrayl)tetraaniline (PyTTA),[S4] zinc 5,10,15,20-tetrakis(p-tetraphenylamino) porphyrin (ZnP),[S5] 1,3,6,8-tetrakis(aminobenzoic)pyrene (TABPy),[S6] 4,4',4''-(1,3,5-triazine-2,4,6-triyl)trianiline (TATTA),[S7] 2,5-diethoxyterephthalohydrazide (DETHz)[S8] were prepared according to the literatures.

Methods

^{1}H NMR spectra were recorded on a JEOL model JNM–LA400 NMR spectrometer, where chemical shifts (δ in ppm) were determined with a residual proton of the solvent as standard. Fourier transform infrared (FT-IR) spectra were recorded on a JASCO model FT-IR-6100 infrared spectrometer. UV-Vis-IR diffuse reflectance spectrum (Kubelka-Munk spectrum) was recorded on a JASCO model V-670 spectrometer equipped with integration sphere model IJN-727. Matrix assisted laser desorption ionization time-of-flight mass (MALDI–TOF–MS) spectra were recorded on an Applied Biosystems BioSpectrometry model Voyager–DE–STR spectrometer in reflector or linear mode using 9-nitroanthracene or dithranol as matrix. Powder X-ray diffraction (PXRD) data were recorded on a Rigaku model RINT Ultima III diffractometer

by depositing powder on glass substrate, from 2θ = 1.5° up to 60° with 0.02° increment. Elemental analysis was performed on a Yanako CHN CORDER MT-6 elemental analyzer.

Nitrogen sorption isotherms were measured at 77 K with a Micromeritics Instrument Corporation model 3Flex surface characterization analyzer. The Brunauer-Emmett-Teller (BET) method was utilized to calculate the specific surface areas. By using the non-local density functional theory (NLDFT) model, the pore volume was derived from the sorption curve.

TATTA-FPBA-COF precursor solution for kinetic studies. A standard solution of TATTA (7.88 mg, 0.022 mmol) and FPBA (10.00 mg, 0.068 mmol) were dissolved in dioxane/mesitylene solution (3:1 v/v, 3 mL) at room temperature, which was sonicated for 1 min. The solution was then filtered (5.00 μm PTFE) to remove any trace residual particulate. The solution was stirred and monitored by absorbance at 1310 nm under different temperatures. Different relative concentrations were prepared based on the concentrations of the above standard solution.

TATTA-FPBA-COF precursor solution for kinetic studies. Because HHTP quickly precipitated in dioxane/mesitylene solution, we used pure dioxane as solvent to prevent the interference of HHTP sediment to the measurement. A standard solution of HHTP (1.81 mg, 0.0056 mmol), FPBA (2.50 mg, 0.017 mmol) and TAPB (1.98 mg, 0.0056 mmol) were dissolved in a dioxane solution (3 mL) at room temperature, which was sonicated for 1 min. The solution was then filtered (5.00 μm PTFE) to remove any trace residual particulate. The solution was stirred and monitored by absorbance at 1310 nm under different temperatures. Different relative concentrations were prepared based on the concentrations of the above standard solution.

The molecular structure and electronic properties of monolayer and stacked X-FPBA-Y COF isomers were determined using the density-functional tight-binding (DFTB) method including Lennard-Jones (LJ) dispersion. The corresponding LJ and crystal stacking energies as well as the HOMO-LUMO energy gaps were computed. The calculations were carried out with the DFTB+ program package version 1.2.[S8] DFTB is an approximate density functional theory method based on the tight binding approach and utilizes an optimized minimal LCAO Slater-type all-valence basis set in combination with a two-center approximation for Hamiltonian matrix elements. The Coulombic interaction between partial atomic charges was determined using the self-consistent charge (SCC) formalism. Lennard-Jones type dispersion was employed in all calculations to describe van der Waals (vdW) and π−stacking interactions. The lattice dimensions were optimized simultaneously with the geometry. Where possible, standard DFTB parameters for X–Y element pair (X, Y = C, N, O, and H) interactions were employed from the mio-0-1 set.[S9] B–X (X = B, C, N, O and H) from the rscc-materials set, F–X (X = C, N, O and H) were taken from the pbc-0-3 set and Cu–X (X = C, H, O and N) parameters were taken from the slko.5425 set.[S10] Parameters for the optimized monolayers are presented below in Table S4.

Using the optimized monomer, different stacking configurations, including eclipsed AA, slipped AA and staggered AB stacking modes, were optimized. In the case of slipped AA structures, structures with slipping in the *a*, *b* and both *a* and *b* directions were optimized. Because the structures of CuPc-FPBA-DETHz COF and CuPc-FPBA-TMBDA COF contain a number of degrees of freedom that are expected to affect the stacking, pre-screening of inclined and serrated stacking motifs was undertaken using UFF4MOF[S11] implemented in GULP.[S12] The third dimension of the lattice, *c* was initialized at 3.5 Å for all planar monolayers and 4.25 Å where the monolayer contained phenyl groups rotated out of the plane of the COF.

Molecular modeling and Pawley refinement were carried out using Reflex, a software package for crystal determination from PXRD pattern, implemented in MS modeling ver 4.4 (Accelrys Inc.).[S13] Unit cell dimension was first manually determined from the observed PXRD peak positions using the coordinates. We performed Pawley refinement to optimize the lattice parameters iteratively until the R_{WP} value converges. The refined parameters of COFs were summarized below in Table S3. Simulated XRD patterns were calculated for eclipsed AA-stacking, slipped AA-stacking, slipped AA-2 and staggered AB stacking modes. After comparing each simulated pattern with experimentally observed pattern, the eclipsed AA, slipped AA and slipped AA-2 staking modes yield a pattern that shows good agreement with the observed XRD curve.

Supplementary Tables

Table S1. The IR vibration bands of the COFs.

**COFs**		**B–O** (cm^{-1})	**C–O** (cm^{-1})	**B–C** (cm^{-1})	**B_3O_3** (cm^{-1})	**C=N** (cm^{-1})
**Hexagonal**	HHTP-FPBA-TATTA	1359	1243	1014	-	1621
	TATTA-FPBA	1363	-	1016	713	1625
**Rhombic**	CuPc-FPBA-ETTA	1342	1287	1087	-	1619
	CuPc-FPBA-PyTTA	1342	1287	1086	-	1618
	CuPc-FPBA-TABPy	1341	1287	1087	-	1618
**Tetragonal**	CuPc-FPBA-ZnP	1342	1284	1084	-	1604
	CuPc-FPBA-TMBDA	1340	1287	1086	-	1633
	CuPc-FPBA-DETHz	1342	1289	1087	-	1609
**Fluoro- and difluoro-substituted linkers**	HHTP-FFPBA-TATTA	1359	1242	1011	-	1621
	HHTP-DFFPBA-TATTA	1360	1243	1011	-	1630
	TATTA-FFPBA	1364	-	1011	714	1625
	TATTA-DFFPBA	1366	-	1011	719	1630

Table S2. Elemental analysis of the COFs.

COFs			C (%)	H (%)	N (%)
**Hexagonal**	HHTP-FPBA-TATTA	Calcd.	70.00	4.70	8.16
		Found	68.81	4.61	8.06
	TATTA-FPBA	Calcd.	72.46	3.91	12.07
		Found	67.95	4.22	11.11
**Rhombic**	CuPc-FPBA-ETTA	Calcd.	66.55	3.38	10.83
		Found	61.21	4.09	10.51
	CuPc-FPBA-PyTTA	Calcd.	69.57	3.39	9.74
		Found	67.83	3.83	9.19
	CuPc-FPBA-TABPy	Calcd.	71.18	3.21	9.22
		Found	66.93	3.66	9.17
**Tetragonal**	CuPc-FPBA-ZnP	Calcd.	65.82	3.19	11.81
		Found	62.15	3.76	12.05
	CuPc-FPBA-TMBDA	Calcd.	65.13	4.00	11.11
		Found	60.33	4.10	11.31
	CuPc-FPBA-DETHz	Calcd.	58.51	3.74	13.00
		Found	56.87	3.92	12.41
**Fluoro- and difluoro-substituted linkers**	HHTP-FFPBA-TATTA	Calcd.	67.07	3.38	7.82
		Found	67.89	3.37	8.04
	HHTP-DFFPBA-TATTA	Calcd.	63.87	2.95	7.45
		Found	60.32	2.55	7.06
	TATTA-FFPBA	Calcd.	67.25	3.22	11.20
		Found	64.62	3.63	11.95
	TATTA-DFFPBA	Calcd.	62.74	2.63	10.45
		Found	58.72	1.86	9.99

Table S3. Pawley refined crystal unit cell parameters.

COFs		Refined unit cell parameters (*a*, *b* and *c* are in Å)	R_{wp} (%)	R_p (%)
**Hexagonal**	HHTP-FPBA-TATTA	a = 33.371, b = 33.615, c = 3.654; α = 93.408°, β = 89.262°, γ = 58.631°	7.19	5.79
	TATTA-FPBA	a = 26.532, b = 25.426, c = 3.490; α = 90.664°, β = 90.168°, γ = 58.144°	4.52	3.06
**Rhombic**	CuPc-FPBA-ETTA	a = 30.622, b = 29.827, c = 3.324; α = 88.281°, β = 90.452°, γ = 86.310°	5.98	4.86
	CuPc-FPBA-PyTTA	a = 35.060, b = 32.206, c = 3.357; α = 81.971°, β = 94.940°, γ = 88.325°	4.09	3.12
	CuPc-FPBA-TABPy	a = 37.761, b = 36.737, c = 3.362; α = 87.496°, β = 90.339°, γ = 87.489°	7.25	5.52
**Tetragonal**	CuPc-FPBA-ZnP	a = 34.033, b = 33.618, c = 3.376; α = 107.11°, β = 88.473°, γ = 93.062°	6.85	5.29
	CuPc-FPBA-TMBDA	a = 36.546, b = 35.738, c = 3.691; α = 96.465°, β = 82.818°, γ = 80.782°	4.76	3.82
	CuPc-FPBA-DETHz	a = 39.728, b = 41.004, c = 3.322; α = 89.376°, β = 89.279°, γ = 77.780°	6.51	5.43
**Fluoro- and difluoro-substituted linkers**	HHTP-FFPBA-TATTA	a = 32.955, b = 33.988, c = 3.463; α = 90.316°, β = 89.427°, γ = 59.240°	8.47	6.85
	HHTP-DFFPBA-TATTA	a = 33.708, b = 33.483, c = 3.451; α = 90.628°, β = 88.879°, γ = 58.753°	8.32	6.69
	TATTA-FFPBA	a = 26.385, b = 25.566, c = 3.504; α = 90.472°, β = 90.695°, γ = 57.880°	4.96	3.58
	TATTA-DFFPBA	a = 26.295, b = 25.543, c = 3.514; α = 90.452°, β = 90.174°, γ = 58.280°	6.08	4.66

Table S4. DFTB⁺ optimised parameters of monolayers

COFs		*a* (Å)	*b* (Å)	*γ* (degree)
**Hexagonal**	HHTP-FPBA-TATTA	33.8	33.8	59
	TATTA-FPBA	26.0	26.0	60
**Rhombic**	CuPc-FPBA-ETTA	30.5	30.0	90
	CuPc-FPBA-PyTTA	35.2	32.0	90
	CuPc-FPBA-TABPy	37.3	37.1	90
**Tetragonal**	CuPc-FPBA-ZnP	34.4	34.4	90
	CuPc-FPBA-TMBDA	35.9	35.9	83
	CuPc-FPBA-DETHz	40.5	40.5	77
**Fluoro- and difluoro-substituted linkers**	HHTP-FFPBA-TATTA	33.8	33.8	59
	HHTP-DFFPBA-TATTA	33.8	33.8	59
	TATTA-FFPBA	26.0	26.0	60
	TATTA-DFFPBA	26.1	26.1	60

Table S5. The total DFTB energies, Lennard-Jones contributions (LJ), and the crystal stacking energies per monolayer as well as the corresponding HOMO-LUMO energy gap for HHTP-FPBA-TATTA COF.

**Stacking modes**	*c* (Å)	**Total DFTB Energy** (a.u.)	**LJ energy** (a.u.)	**Crystal stacking energy per unit cell per layer** (kcal mol^{-1})	**HOMO-LUMO gap** (eV)
**Monolayer**		-153.330676	0.6308		2.481
**Eclipsed AA**	3.50	-306.929054	0.9808	-88.83	2.203
**Slipped AA (0.4 Å in *a*, 1.1 Å in *b*)**	3.40	-306.941783	0.9785	-89.84	2.385
**Slipped AA-2 (1.0 Å in *b*)**	3.47	-306.941518	0.9766	-90.35	2.303
**Staggered AB**	3.23	-306.7881792	1.1377	-36.32	2.274

Table S6. The total DFTB energies, Lennard-Jones contributions (LJ), and the crystal stacking energies per monolayer as well as the corresponding HOMO-LUMO energy gap for TATTA-FPBA COF.

**Stacking modes**	*c* (Å)	**Total DFTB Energy** (a.u.)	**LJ energy** (a.u.)	**Crystal stacking energy per unit cell per layer** (kcal mol^{-1})	**HOMO-LUMO gap** (eV)
**Monolayer**		-110.236780	0.4531		2.473
**Eclipsed AA**	3.54	-220.669255	0.7027	-63.88	2.180
**Slipped AA (0.4 Å in *a*, 1.0 Å in *b*)**	3.43	-220.674051	0.7032	-63.73	2.345
**Slipped AA-2 (1.4 Å in *a*, 0.8 Å in *b*)**	3.44	-220.674295	0.7037	-63.57	2.327
**Staggered AB**	3.03	-220.5826881	0.8020	-32.72	2.314

Table S7. The total DFTB energies, Lennard-Jones contributions (LJ), and the crystal stacking energies per monolayer as well as the corresponding HOMO-LUMO energy gap for CuPc-FPBA-ETTA COF.

**Stacking modes**	***c*** (Å)	**Total DFTB Energy** (a.u.)	**LJ energy** (a.u.)	**Crystal stacking energy per unit cell per layer** (kcal mol^{-1})	**HOMO-LUMO gap** (eV)
**Monolayer**		-226.493345	0.8765		0.134
**Eclipsed AA**	3.86	-453.242423	1.4239	-80.24	0.079
**Slipped AA (1.0 Å in *a*)**	3.75	-453.245247	1.4376	-81.12	0.038
**Slipped AA-2 (0.9 Å in *a*)**	3.75	-453.247356	1.4419	-81.79	0.045
**Staggered AB**	3.25	-453.1924803	1.5519	-64.57	0.140

Table S8. The total DFTB energies, Lennard-Jones contributions (LJ), and the crystal stacking energies per monolayer as well as the corresponding HOMO-LUMO energy gap for CuP-FPBA-PyTTA COF.

**Stacking modes**	***c*** (Å)	**Total DFTB Energy** (a.u.)	**LJ energy** (a.u.)	**Crystal stacking energy per unit cell per layer** (kcal mol^{-1})	**HOMO-LUMO gap** (eV)
**Monolayer**		-252.761685	1.0140		1.408
**Eclipsed AA**	3.60	-505.940051	1.5772	-130.74	0.761
**Slipped AA (0.7 Å in *a* and 0.4 Å in *b*)**	3.57	-505.945563	1.5775	-132.46	0.822
**Slipped AA-2 (0.5-Å in *a* and *b*)**	3.57	-505.9399809	1.5818	-130.71	0.850
**Staggered AB**	3.55	-505.920864	1.6137	-124.72	1.349

Table S9. The total DFTB energies, Lennard-Jones contributions (LJ), and the crystal stacking energies per monolayer as well as the corresponding HOMO-LUMO energy gap for CuPc-FPBA-TABPy COF.

**Stacking modes**	***c*** (Å)	**Total DFTB Energy** (a.u.)	**LJ energy** (a.u.)	**Crystal stacking energy per unit cell per layer** (kcal mol^{-1})	**HOMO-LUMO gap** (eV)
**Monolayer**		-266.302368	1.0475		0.135
**Eclipsed AA**	3.54	-533.082259	1.6064	-149.82	0.130
**Slipped AA (1.1 Å in *a*)**	3.44	-533.090023	1.6052	-152.26	0.097
**Slipped AA-2 (1.1 Å in *b*)**	3.44	-533.089074	1.6061	-151.96	0.100
**Staggered AB**	3.11	-532.8097128	1.8939	-64.31	0.132

Table S10. The total DFTB energies, Lennard-Jones contributions (LJ), and the crystal stacking energies per monolayer as well as the corresponding HOMO-LUMO energy gap for CuPc-FPBA-ZnP COF.

**Stacking modes**	***c*** (Å)	**Total DFTB Energy** (a.u.)	**LJ energy** (a.u.)	**Crystal stacking energy per unit cell per layer** (kcal mol^{-1})	**HOMO-LUMO gap** (eV)
**Monolayer**		-274.327312	1.0468		0.134
**Eclipsed AA**	3.67	-548.994126	1.6939	-125.39	0.126
**Slipped AA (0.1 Å in *a* and *b*)**	3.68	-548.996176	1.6882	-127.18	0.125
**Reversed AA (CuPc aligned on top of ZnP)**	3.84	-548.982703	1.7405	-110.77	0.103
**Staggered AB**	3.25	-548.8672518	1.8835	-65.90	0.142

Table S11. The total DFTB energies, Lennard-Jones contributions (LJ), and the crystal stacking energies per monolayer as well as the corresponding HOMO-LUMO energy gap for CuP-FPBA-TMBDA COF.

**Stacking modes**	***c*** (Å)	**Total DFTB Energy** (a.u.)	**LJ energy** (a.u.)	**Crystal stacking energy per unit cell per layer** (kcal mol^{-1})	**HOMO-LUMO gap** (eV)
**Monolayer**		-219.255329	0.8643		1.424
**Eclipsed AA**	3.72	-438.862562	1.3555	-110.41	1.088
**Slipped AA (0.7 Å in *a* and *b*)**	3.59	-438.882699	1.3441	-116.73	1.119
**Slipped AA-2 (0.9 Å in *a* and *b*)**	3.58	-438.874737	1.3487	-114.23	1.172
**Staggered AB**	3.62	-438.642181	1.5831	-41.27	1.297

Table S12. The total DFTB energies, Lennard-Jones contributions (LJ), and the crystal stacking energies per monolayer as well as the corresponding HOMO-LUMO energy gap for CuP-FPBA-DETHz COF.

**Stacking modes**	***c*** (Å)	**Total DFTB Energy** (a.u.)	**LJ energy** (a.u.)	**Crystal stacking energy per unit cell per layer** (kcal mol^{-1})	**HOMO-LUMO gap** (eV)
**Monolayer**		-263.523306	0.9431		1.423
**Eclipsed AA**	3.65	-527.381120	1.4931	-104.95	1.216
**Slipped AA (1.9 Å in *a*)**	3.33	-527.475234	1.4644	-134.48	1.299
**Slipped AA-2 (2.8 Å in *a*, 2.4 Å in b)**	3.30	-527.457284	1.4761	-128.85	1.232
**Staggered AB**	3.49	-527.236944	1.7069	-59.72	1.420

Table S13. The total DFTB energies, Lennard-Jones contributions (LJ), and the crystal stacking energies per monolayer as well as the corresponding HOMO-LUMO energy gap for HHTP-FFPBA-TATTA COF.

**Stacking modes**	***c*** (Å)	**Total DFTB Energy** (a.u.)	**LJ energy** (a.u.)	**Crystal stacking energy per unit cell per layer** (kcal mol^{-1})	**HOMO-LUMO gap** (eV)
**Monolayer**		-165.341181	0.6323		2.462
**Eclipsed AA**	3.50	-330.953015	0.9815	-88.83	2.089
**Slipped AA (0.4 Å in a, 1.0 Å in b)**	3.40	-330.966091	0.9783	-89.84	2.244
**Slipped AA-2 (0.4 Å in a, 1.2 Å in b)**	3.45	-330.968944	0.9766	-90.35	2.190
**Staggered AB**	3.15	-330.7992835	1.1488	-36.32	2.131

Table S14. The total DFTB energies, Lennard-Jones contributions (LJ), and the crystal stacking energies per monolayer as well as the corresponding HOMO-LUMO energy gap for HHTP-DFFPBA-TATTA COF.

**Stacking modes**	***c*** (Å)	**Total DFTB Energy** (a.u.)	**LJ energy** (a.u.)	**Crystal stacking energy per unit cell per layer** (kcal mol^{-1})	**HOMO-LUMO gap** (eV)
**Monolayer**		-177.321436	0.6332		2.405
**Eclipsed AA**	3.50	-354.911001	0.9814	-89.40	2.111
**Slipped AA (0.5 Å in *a*, 1.2 Å in *b*)**	3.39	-354.931451	0.9799	-89.86	2.315
**Slipped AA-2 (1.3 Å in *a*, 1.1 Å in *b*)**	3.44	-354.928434	0.9776	-90.61	2.265
**Staggered AB**	3.17	-354.7714868	1.1418	-39.09	2.231

Table S15. The total DFTB energies, Lennard-Jones contributions (LJ), and the crystal stacking energies per monolayer as well as the corresponding HOMO-LUMO energy gap for TATTA-FFPBA COF.

**Stacking modes**	***c*** (Å)	**Total DFTB Energy** (a.u.)	**LJ energy** (a.u.)	**Crystal stacking energy per unit cell per layer** (kcal mol^{-1})	**HOMO-LUMO gap** (eV)
**Monolayer**		-122.240392	0.4540		2.295
**Eclipsed AA**	3.53	-244.677445	0.7016	-64.73	1.915
**Slipped AA (0.4 Å in *a*, 1.0 Å in *b*)**	3.42	-244.683682	0.7026	-64.43	2.136
**Slipped AA-2 (1.0 Å in *a*, 0.2 Å in *b*)**	3.44	-244.683527	0.7024	-64.47	2.070
**Staggered AB**	3.03	-244.5895932	0.8032	-32.84	2.029

Table S16. The total DFTB energies, Lennard-Jones contributions (LJ), and the crystal stacking energies per monolayer as well as the corresponding HOMO-LUMO energy gap for TATTA-DFFPBA COF.

**Stacking modes**	***c*** (Å)	**Total DFTB Energy** (a.u.)	**LJ energy** (a.u.)	**Crystal stacking energy per unit cell per layer** (kcal mol^{-1})	**HOMO-LUMO gap** (eV)
**Monolayer**		-134.220818	0.4574		2.413
**Eclipsed AA**	3.55	-268.644128	0.6998	-67.47	1.954
**Slipped AA (0.4 Å in *a*, 1.0 Å in *b*)**	3.42	-268.651707	0.7012	-67.02	2.167
**Slipped AA-2 (1.5 Å in *a*, 1.0 Å in *b*)**	3.43	-268.652027	0.7011	-67.06	2.147
**Staggered AB**	3.08	-268.5529319	0.8020	-35.40	2.143

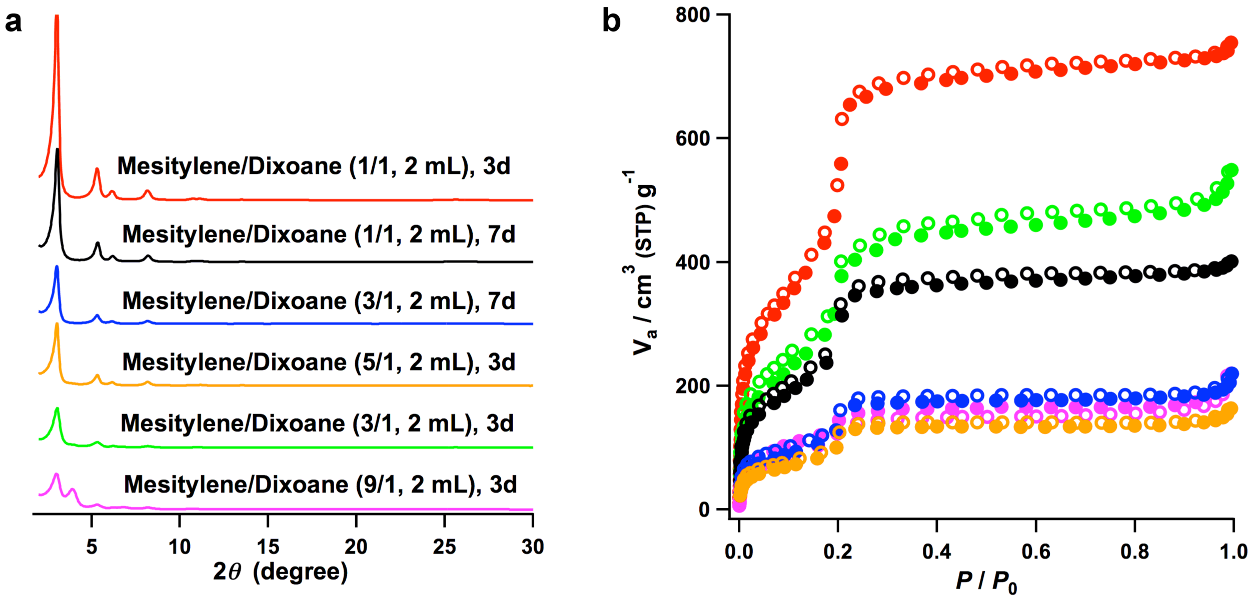

Figure S1. a, XRD patterns of the HHTP-FPBA-TATTA COF samples prepared under different conditions. **b**, Nitrogen sorption curves of the HHTP-FPBA-TATTA COF samples prepared under different conditions (filled circles: adsorption; open circles: desorption (the same for Figures S1-S12); red: Mesitylene/Dioxane = 1/1, 2 mL, 3 d; green: Mesitylene/Dioxane = 3/1, 2 mL, 3 d; black: Mesitylene/Dioxane = 3/1, 2 mL, 7 d; blue: Mesitylene/Dioxane = 1/1, 2 mL, 7d; magenta: Mesitylene/Dioxane = 9/1, 2 mL, 3 d; orange: Mesitylene/Dioxane = 5/1, 2 mL, 3 d).

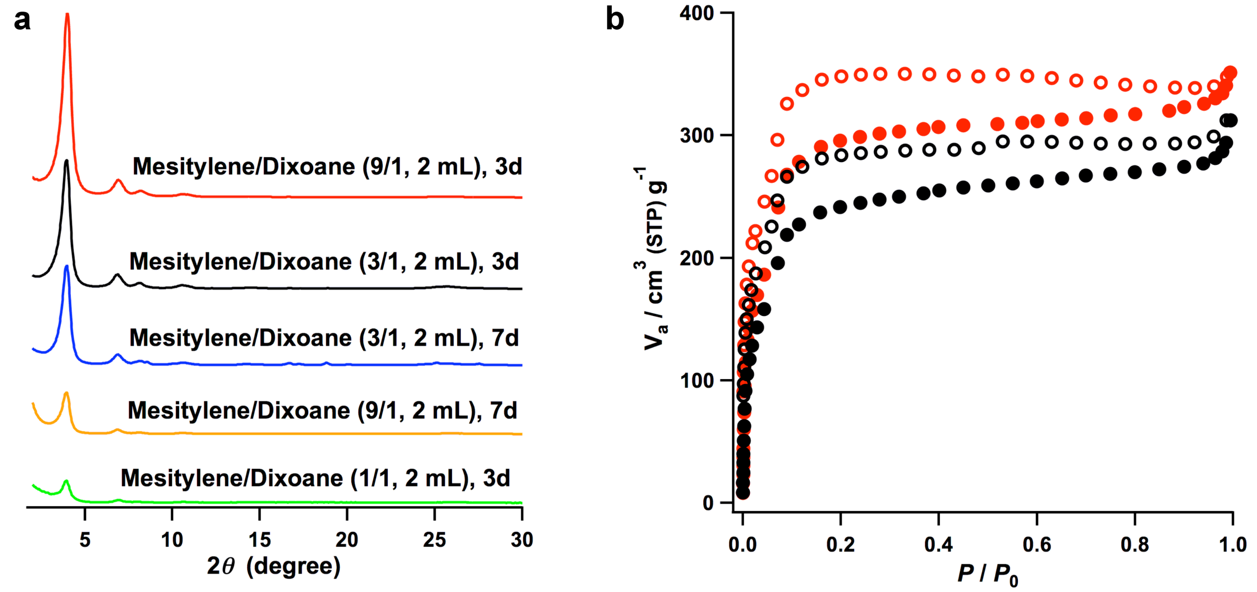

Figure S2. a, XRD patterns of the TATTA-FPBA COF samples prepared under different conditions. **b**, Nitrogen sorption curves of the TATTA-FPBA COF samples prepared under different conditions (red: Mesitylene/Dioxane = 9/1, 2 mL, 3 d; black: Mesitylene/Dioxane = 3/1, 2 mL, 3 d).

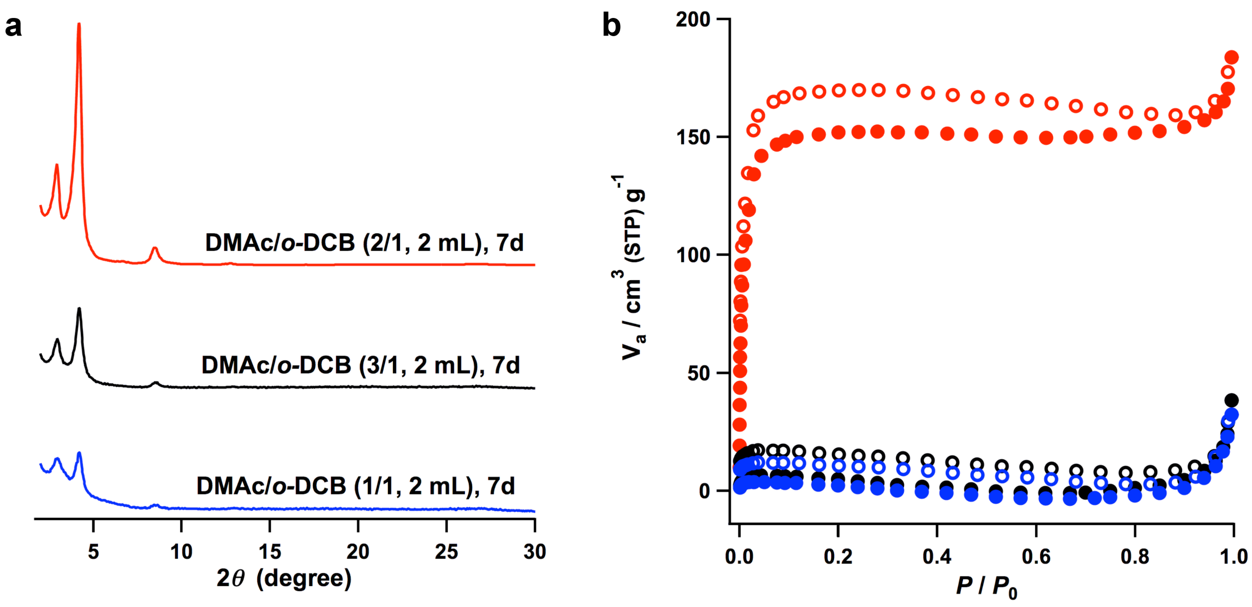

Figure S3. **a**, XRD patterns of the CuPc-FPBA-ETTA COF samples prepared under different conditions. **b**, Nitrogen sorption curves of the CuPc-FPBA-ETTA COF samples prepared under different conditions (red: DMAc/*o*-DCB = 2/1, 2 mL, 7 d; black: DMA*c/*o-DCB = 3/1, 2 mL, 7 d; blue: DMAc/*o*-DCB = 1/1, 2 mL, 7 d).

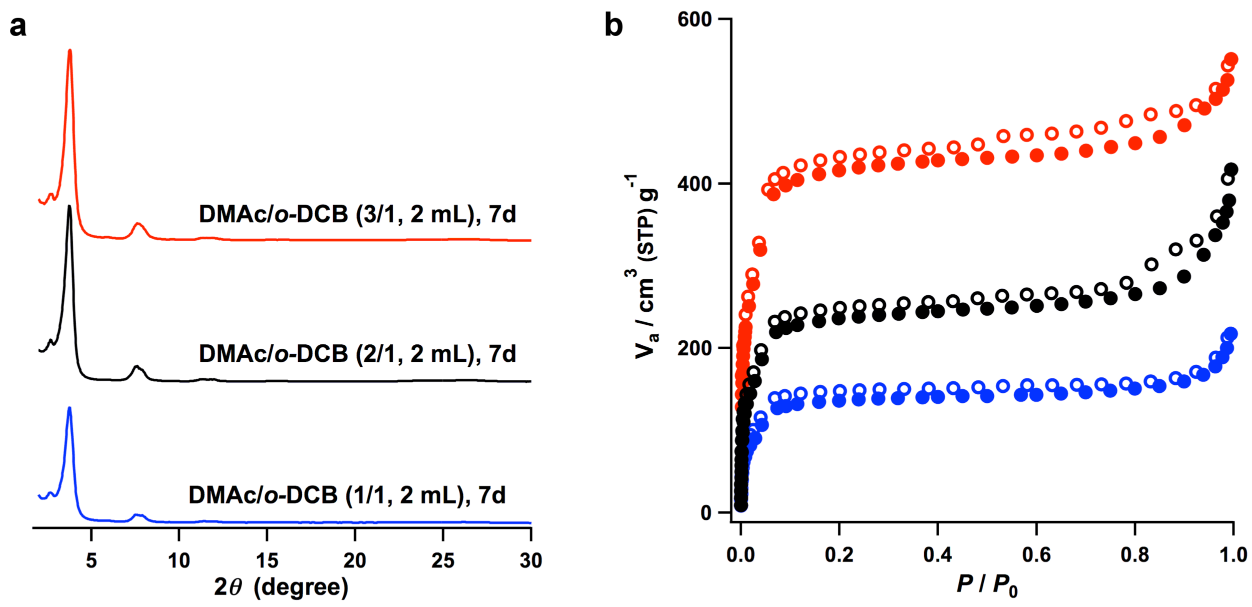

Figure S4. **a**, XRD patterns of the CuPc-FPBA-PyTTA COF samples prepared under different conditions. **b**, Nitrogen sorption curves of the CuPc-FPBA-PyTTA COF samples prepared under different conditions (red: DMAc/*o*-DCB = 2/1, 2 mL, 7 d; black: DMAc/*o*-DCB = 3/1, 2 mL, 7 d; blue: DMAc/*o*-DCB = 1/1, 2 mL, 7 d).

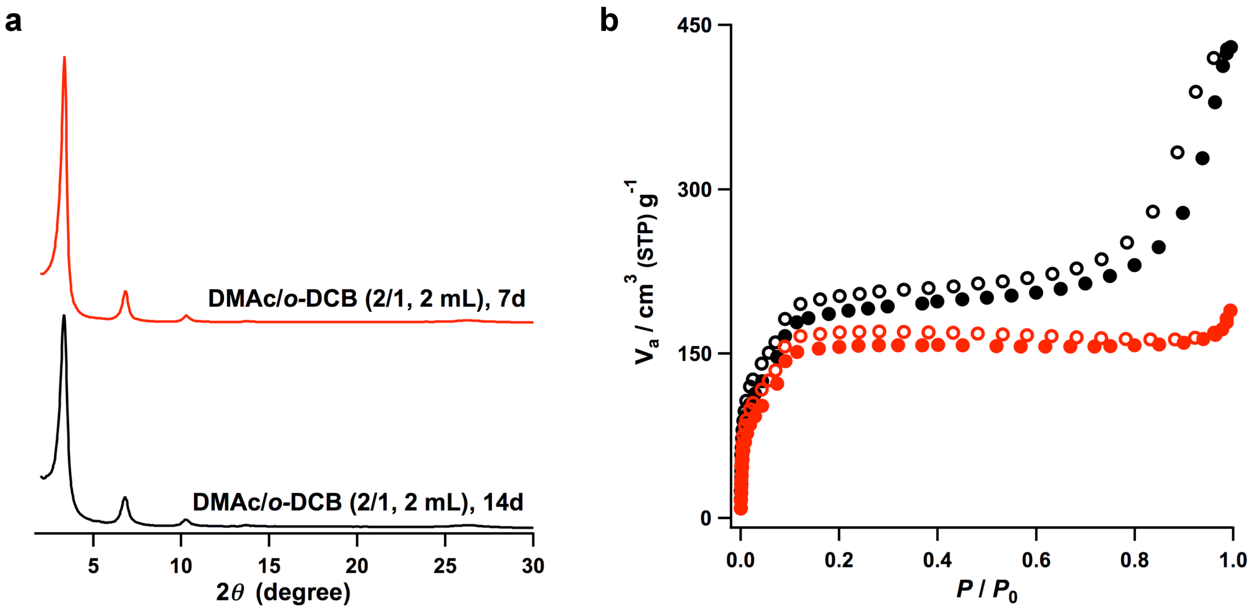

Figure S5. **a**, XRD patterns of the CuPc-FPBA-TABPy COF samples prepared under different conditions. **b**, Nitrogen sorption curves of the CuPc-FPBA-TABPy COF samples prepared under different conditions (red: DMAc/*o*-DCB = 2/1, 2 mL, 7 d; black: DMAc/*o*-DCB = 2/1, 2 mL, 14 d).

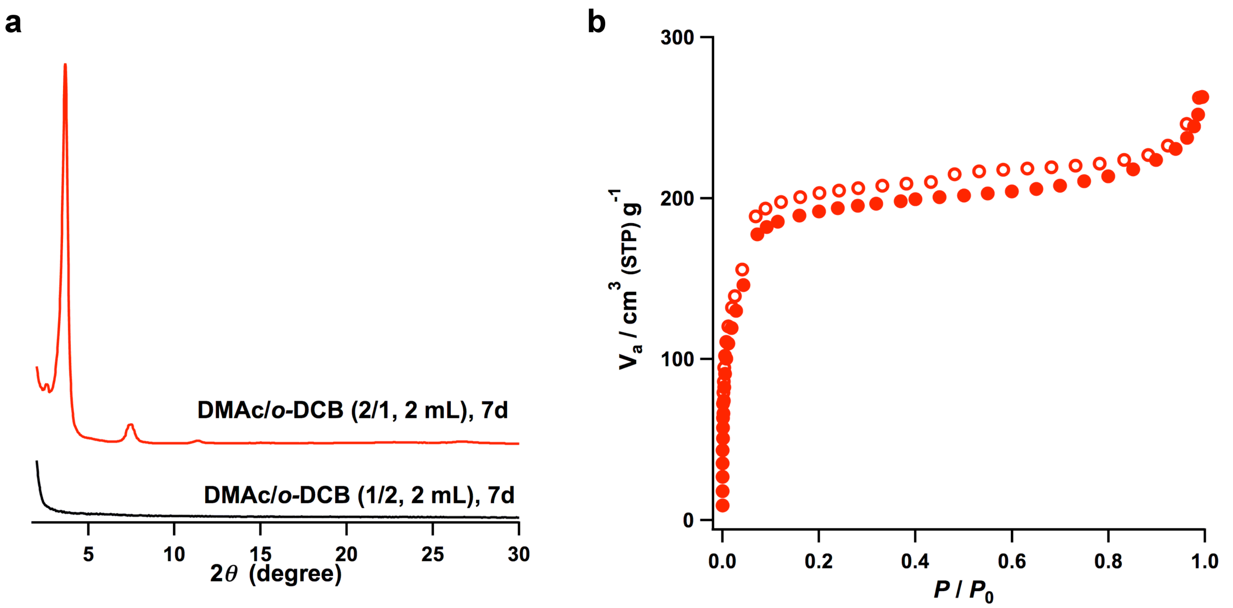

Figure S6. **a**, XRD patterns of the CuPc-FPBA-ZnP COF samples prepared under different conditions (red: DMAc/*o*-DCB = 2/1, 2 mL, 7 d; black: DMAc/*o*-DCB = 1/2, 2 mL, 7 d). **b**, Nitrogen sorption curves of the CuPc-FPBA-ZnP COF samples prepared (DMAc/*o*-DCB = 2/1, 2 mL, 7 d).

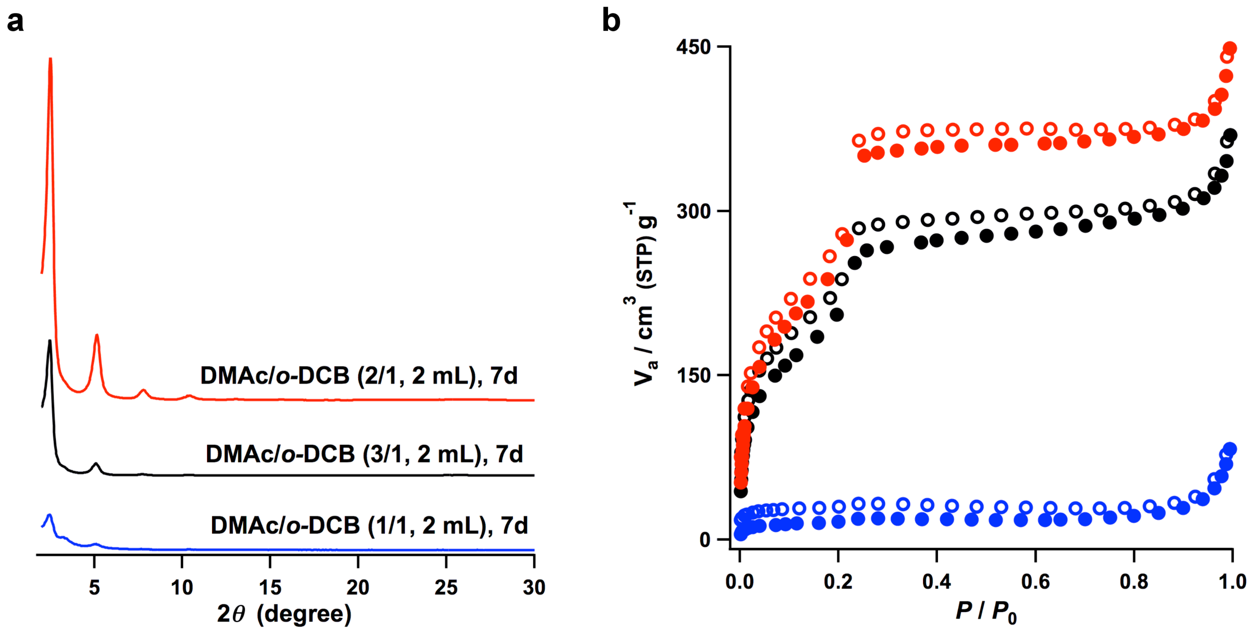

Figure S7. a, XRD patterns of the CuPc-FPBA-TMBDA COF samples prepared under different conditions. **b**, Nitrogen sorption curves of the CuPc-FPBA-TMBDA COF samples prepared under different conditions (red: DMAc/*o*-DCB = 2/1, 2 mL, 7 d; black: DMAc/*o*-DCB = 3/1, 2 mL, 7 d; blue: DMAc/*o*-DCB = 1/1, 2 mL, 7 d).

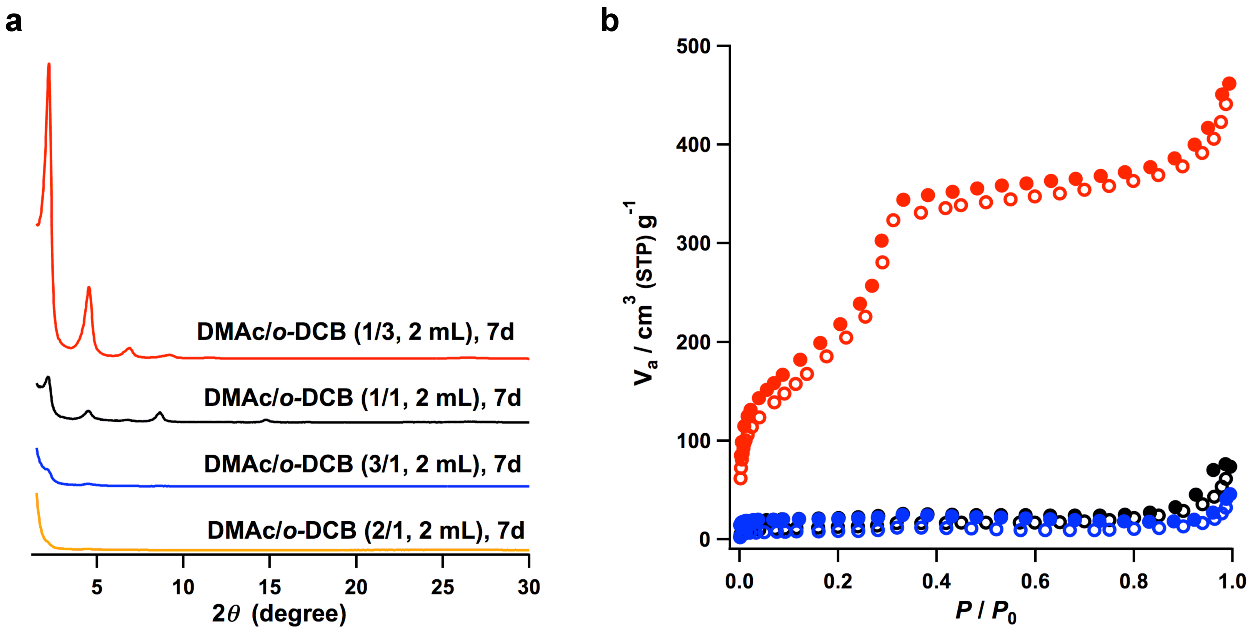

Figure S8. a, XRD patterns of the CuPc-FPBA-DETHz COF samples prepared under different conditions. **b**, Nitrogen sorption curves of the CuPc-FPBA-DETHz COF samples prepared under different conditions (red: DMAc/*o*-DCB = 1/3, 2 mL, 7 d; black: DMAc/*o*-DCB = 1/1, 2 mL, 7 d; blue: DMAc/*o*-DCB = 3/1, 2 mL, 7 d).

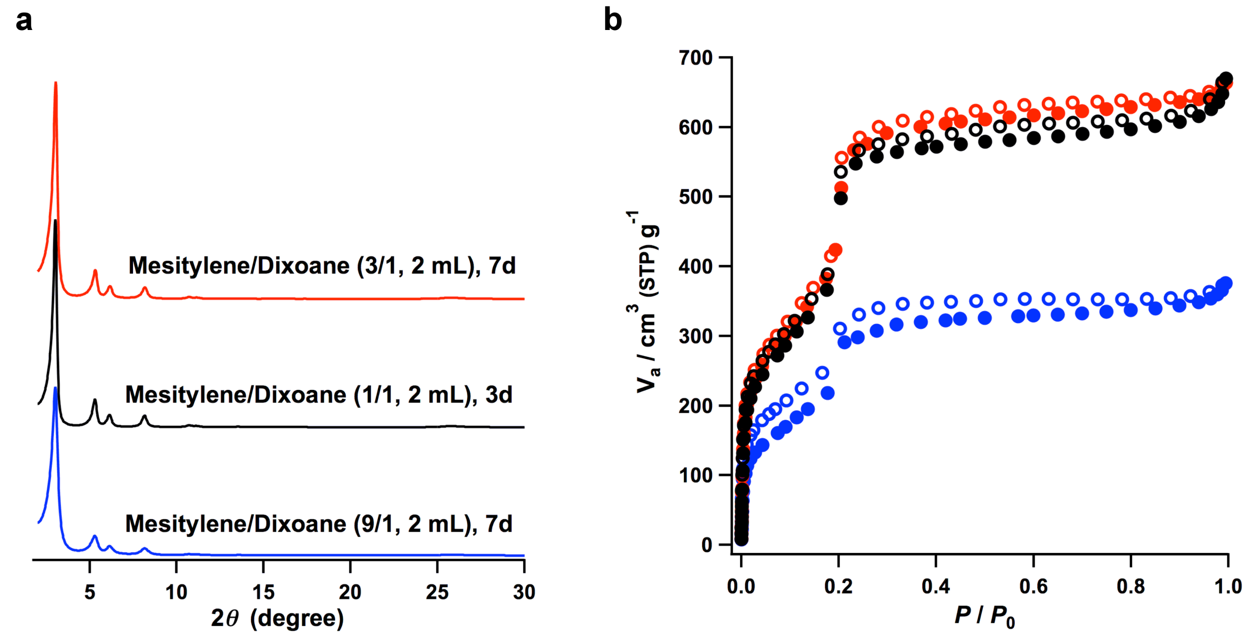

Figure S9. **a**, XRD patterns of the HHTP-FFPBA-TATTA COF samples prepared under different conditions. **b**, Nitrogen sorption curves of the HHTP-FFPBA-TATTA COF samples prepared under different conditions (red: Mesitylene/Dioxane = 3/1, 2 mL, 7 d; black: Mesitylene/Dioxane = 1/1, 2 mL, 3 d; blue: Mesitylene/Dioxane = 9/1, 2 mL, 7 d).

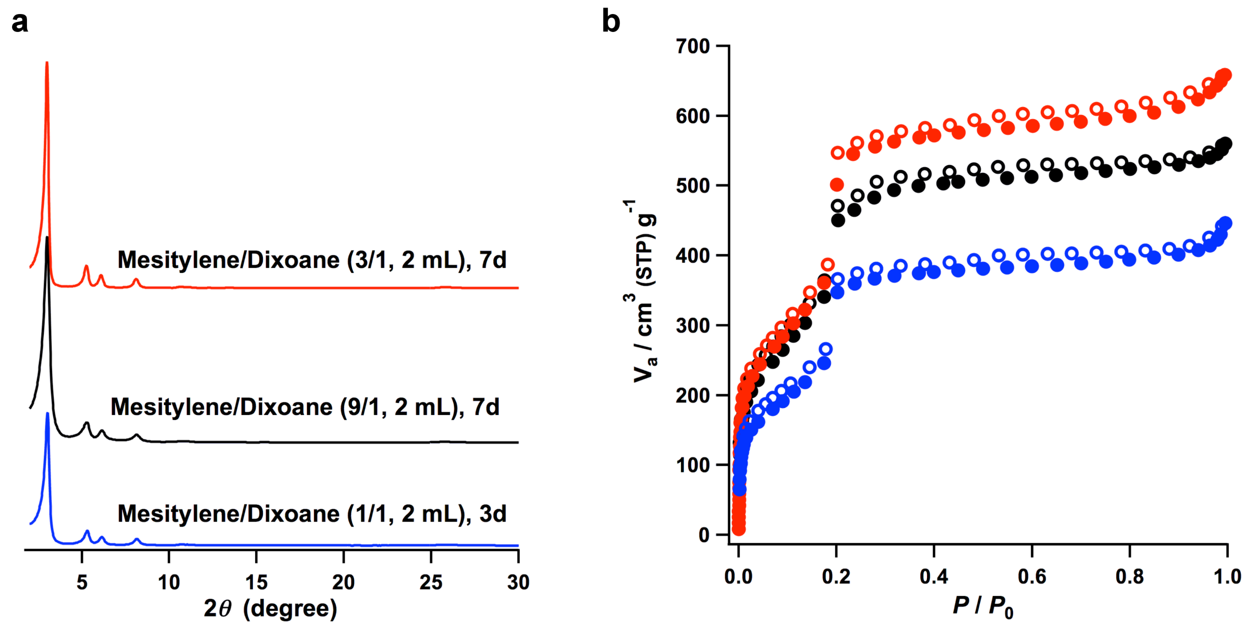

Figure S10. **a**, XRD patterns of the HHTP-DFFPBA-TATTA COF samples prepared under different conditions. **b**, Nitrogen sorption curves of the HHTP-DFFPBA-TATTA COF samples prepared under different conditions (red: Mesitylene/Dioxane = 3/1, 2 mL, 7 d; black: Mesitylene/Dioxane = 9/1, 2 mL, 7 d; blue: Mesitylene/Dioxane = 1/1, 2 mL, 3 d).

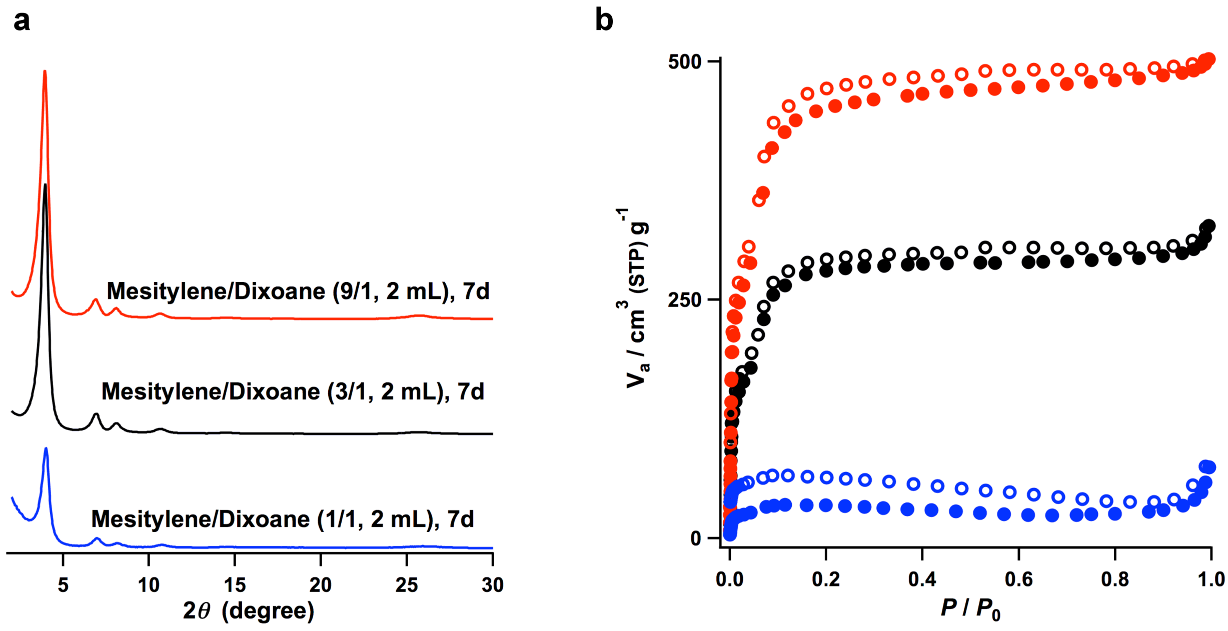

Figure S11. a, XRD patterns of the TATTA-FFPBA COF samples prepared under different conditions. **b**, Nitrogen sorption curves of the TATTA-FFPBA COF samples prepared under different conditions (red: Mesitylene/Dioxane = 9/1, 2 mL, 7 d; black: Mesitylene/Dioxane = 3/1, 2 mL, 7 d; blue: Mesitylene/Dioxane = 1/1, 2 mL, 7 d).

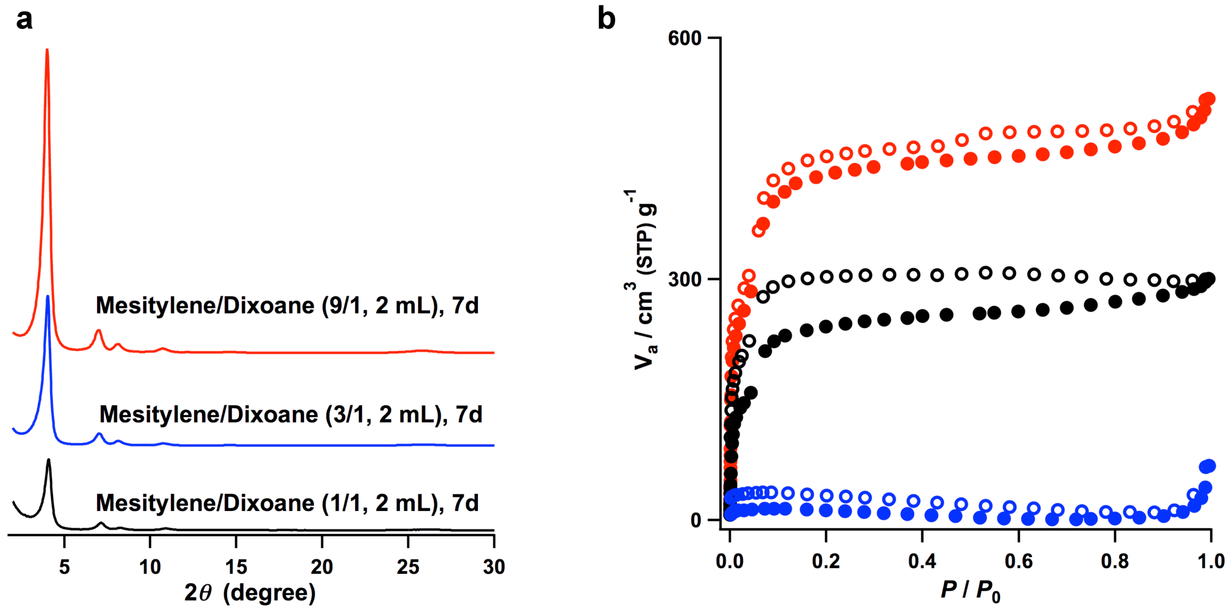

Figure S12. a, XRD patterns of the TATTA-DFFPBA COF samples prepared under different conditions. **b**, Nitrogen sorption curves of the TATTA-DFFPBA COF samples prepared under different conditions (red: Mesitylene/Dioxane = 9/1, 2 mL, 7 d; black: Mesitylene/Dioxane = 3/1, 2 mL, 7 d; blue: Mesitylene/Dioxane = 1/1, 2 mL, 7 d).

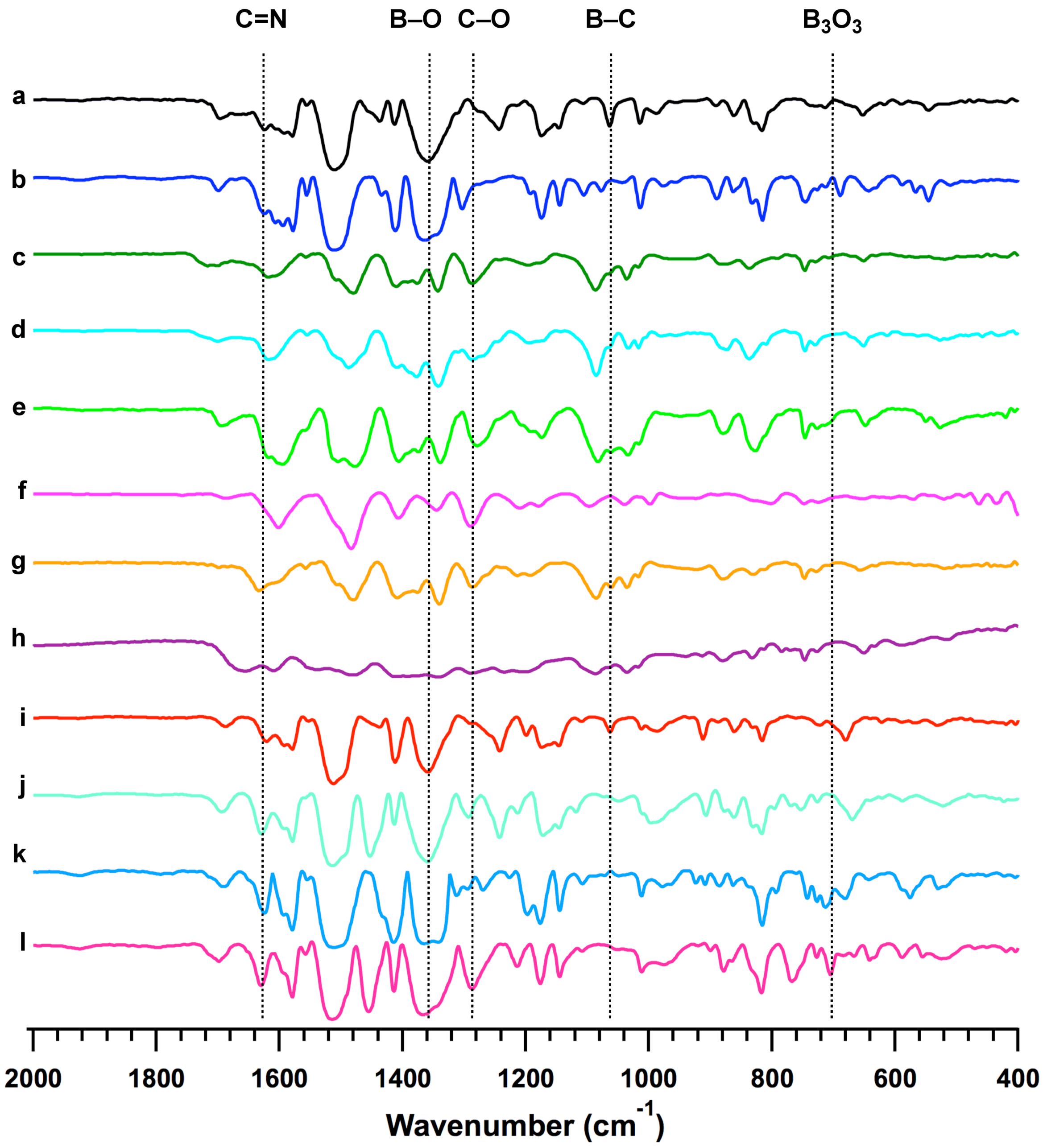

Figure S13. FT-IR spectra of (a) HHTP-FPBA-TATTA COF, (b) TATTA-FPBA COF, (c) CuPc-FPBA-ETTA COF, (d) CuPc-FPBA-PyTTA COF, (e) CuPc-FPBA-TABPy COF, (f) CuPc-FPBA-ZnP COF, (g) CuPc-FPBA-TMBDA COF, (h) CuPc-FPBA-DETHz COF, (i) HHTP-FFPBA-TATTA COF, (j) HHTP-DFFPBA-TATTA COF, (k) TATTA-FFPBA COF and (k) TATTA-DFFPBA COF.

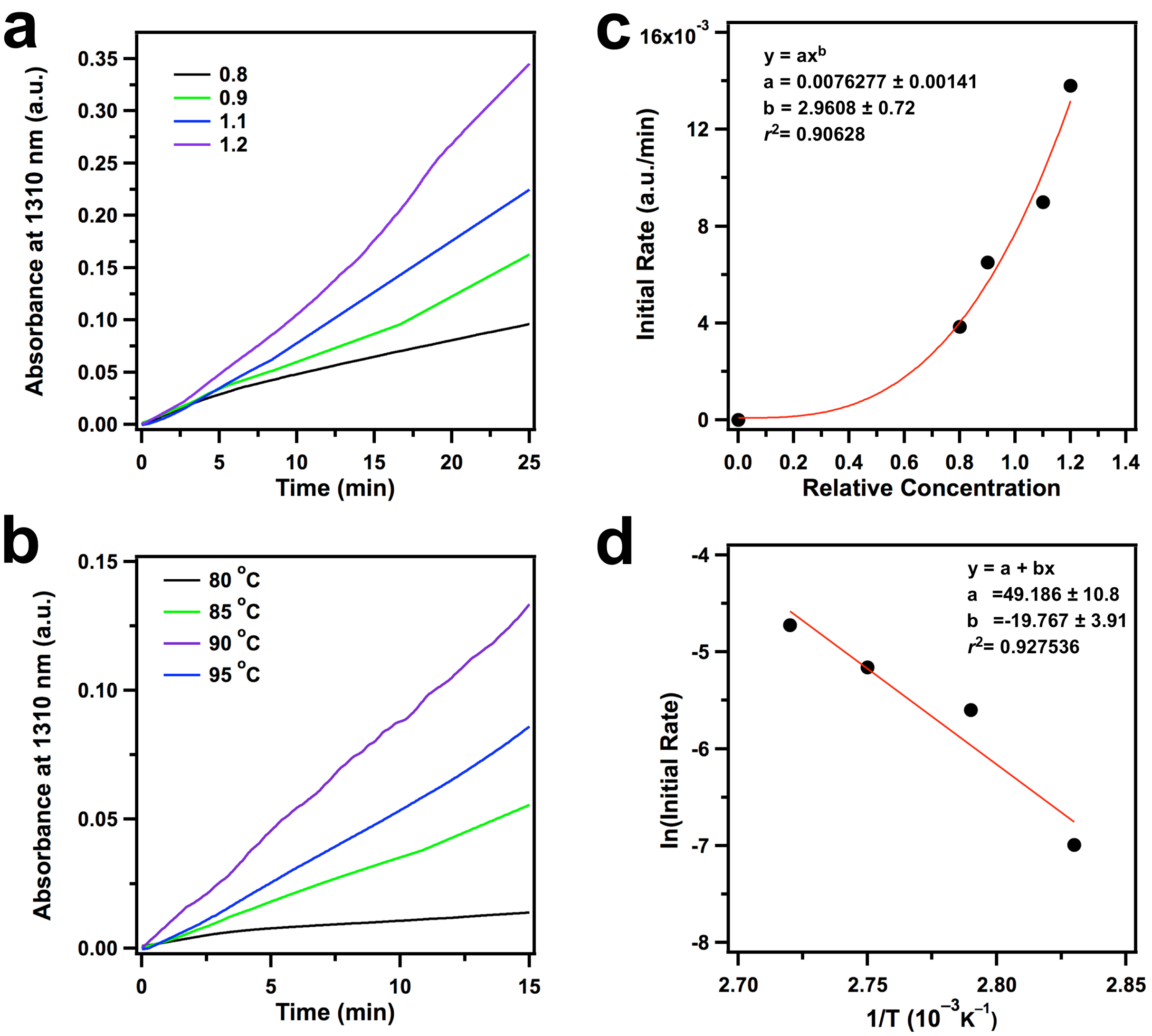

Figure S14. (a) Time-dependent absorbance change of the reaction to prepare the HHTP-FPBA-TATTA COF with different relative concentrations of monomers (standard concentration, 1.86 mM TATTA, 1.86 mM HHTP and 5.58 mM FPBA) at 90 °C. (b) Time-dependent absorbance change of the reaction to prepare the HHTP-FPBA-TATTA COF at different temperatures. (c) Plot of initial rate versus the relative concentration of monomers. (d) Pseudo-Arrhenius plot.

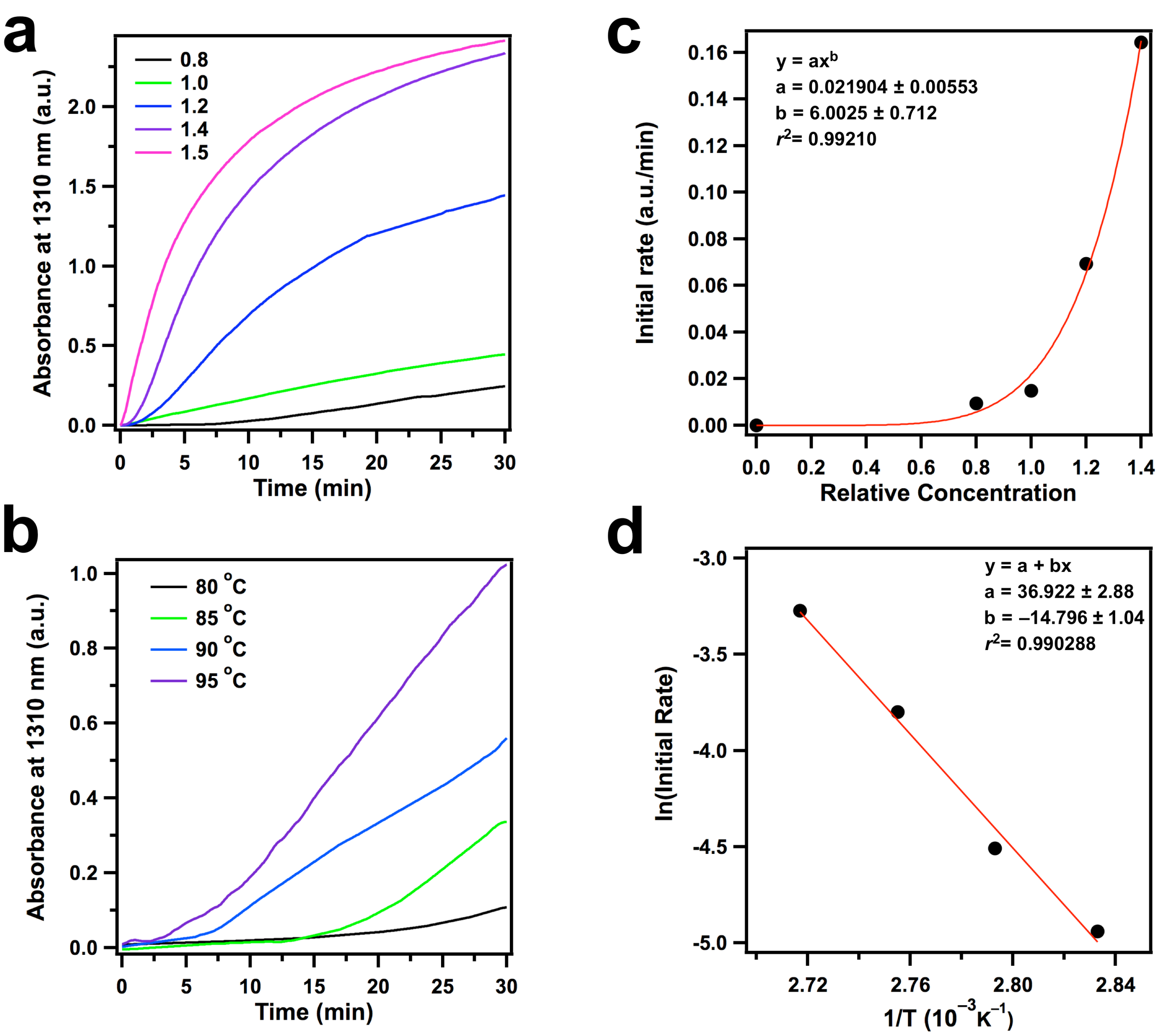

Figure S15. Figure S14. (a) Time-dependent absorbance change of the reaction to prepare the TATTA-FPBA COF with different relative concentrations of monomers (standard concentration, 7.41 mM TATTA and 22.23 mM FPBA) at 90 °C. (b) Time-dependent absorbance change of the reaction to prepare the TATTA-FPBA COF at different temperatures. (c) Plot of initial rate versus the relative concentration of monomers. (d) Pseudo-Arrhenius plot.

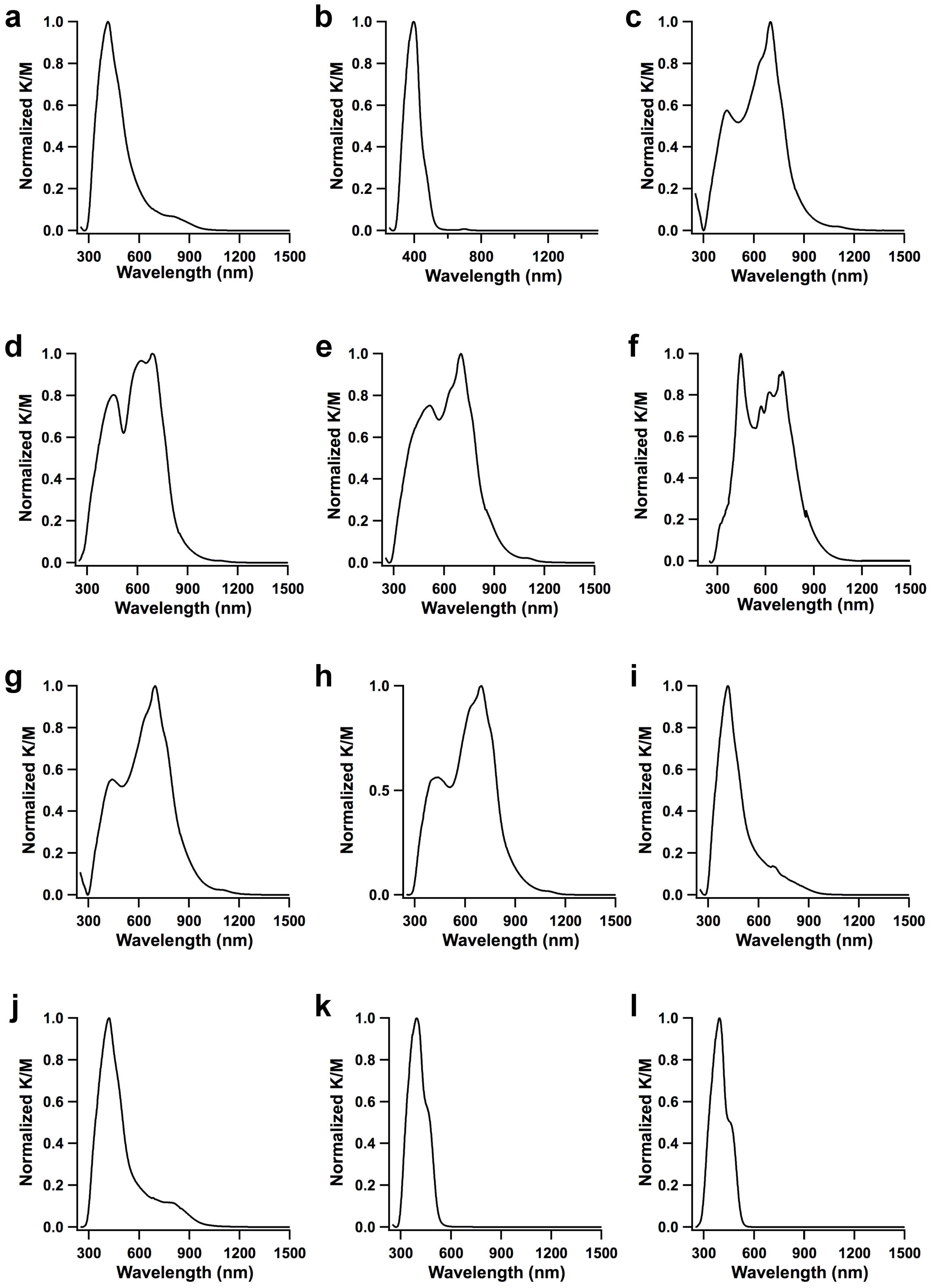

Figure S16. Solid-state electronic absorption spectra of (a) HHTP-FPBA-TATTA COF, (b) TATTA-FPBA COF, (c) CuPc-FPBA-ETTA COF, (d) CuPc-FPBA-PyTTA COF, (e) CuPc-FPBA-TABPy COF, (f) CuPc-FPBA-ZnP COF, (g) CuPc-FPBA-TMBDA COF, (h) CuPc-FPBA-DETHz COF, (i) HHTP-FFPBA-TATTA COF, (j) HHTP-DFFPBA-TATTA COF, (k) TATTA-FFPBA COF and (l) TATTA-DFFPBA COF.

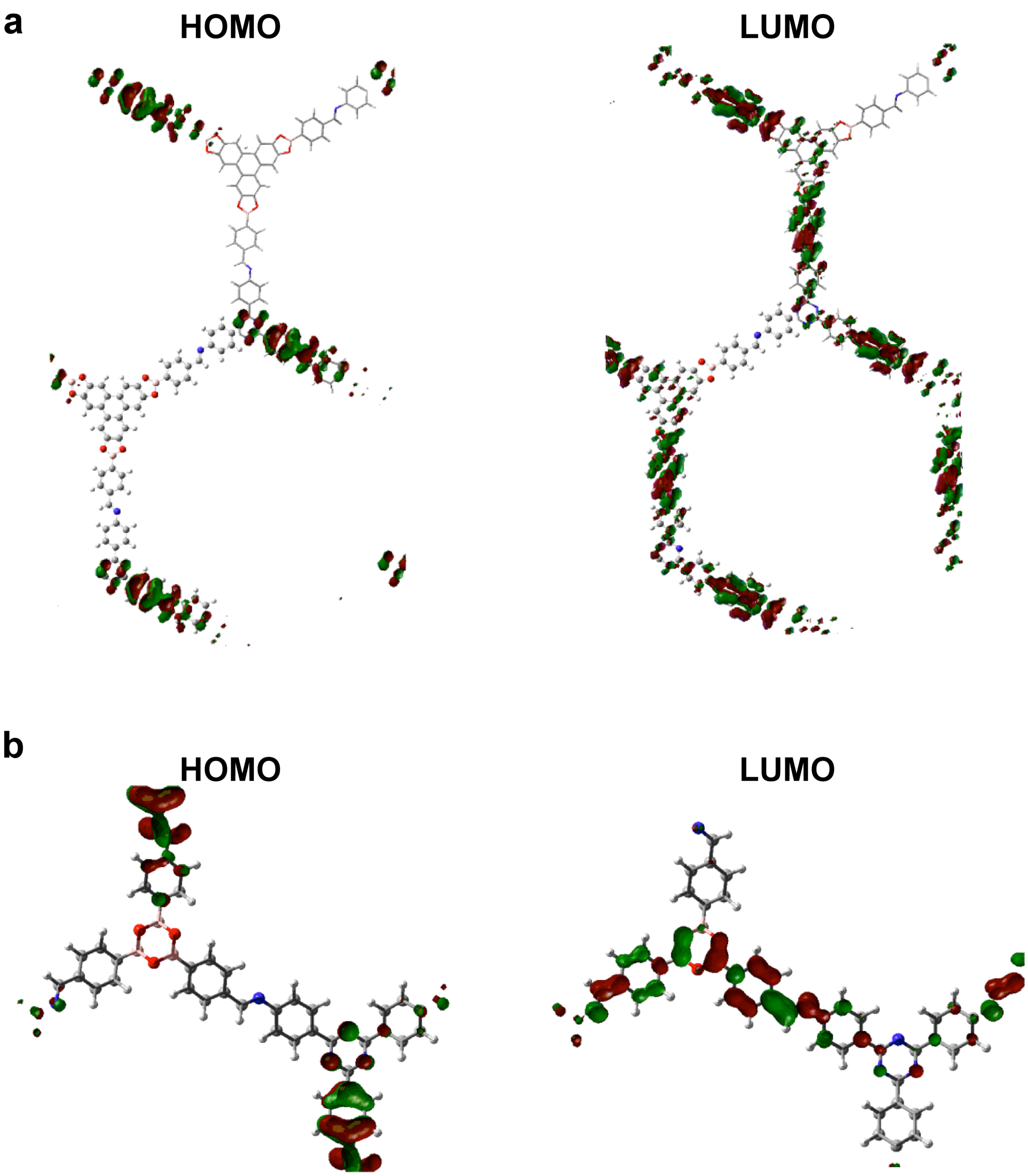

Figure S17. Frontier molecular orbitals of (a) the HHTP-FPBA-TATTA COF and (b) the TATTA- FPBA COF. Bottom layer is depicted using ball-and-stick mode, top layer is shown using the tube representation.

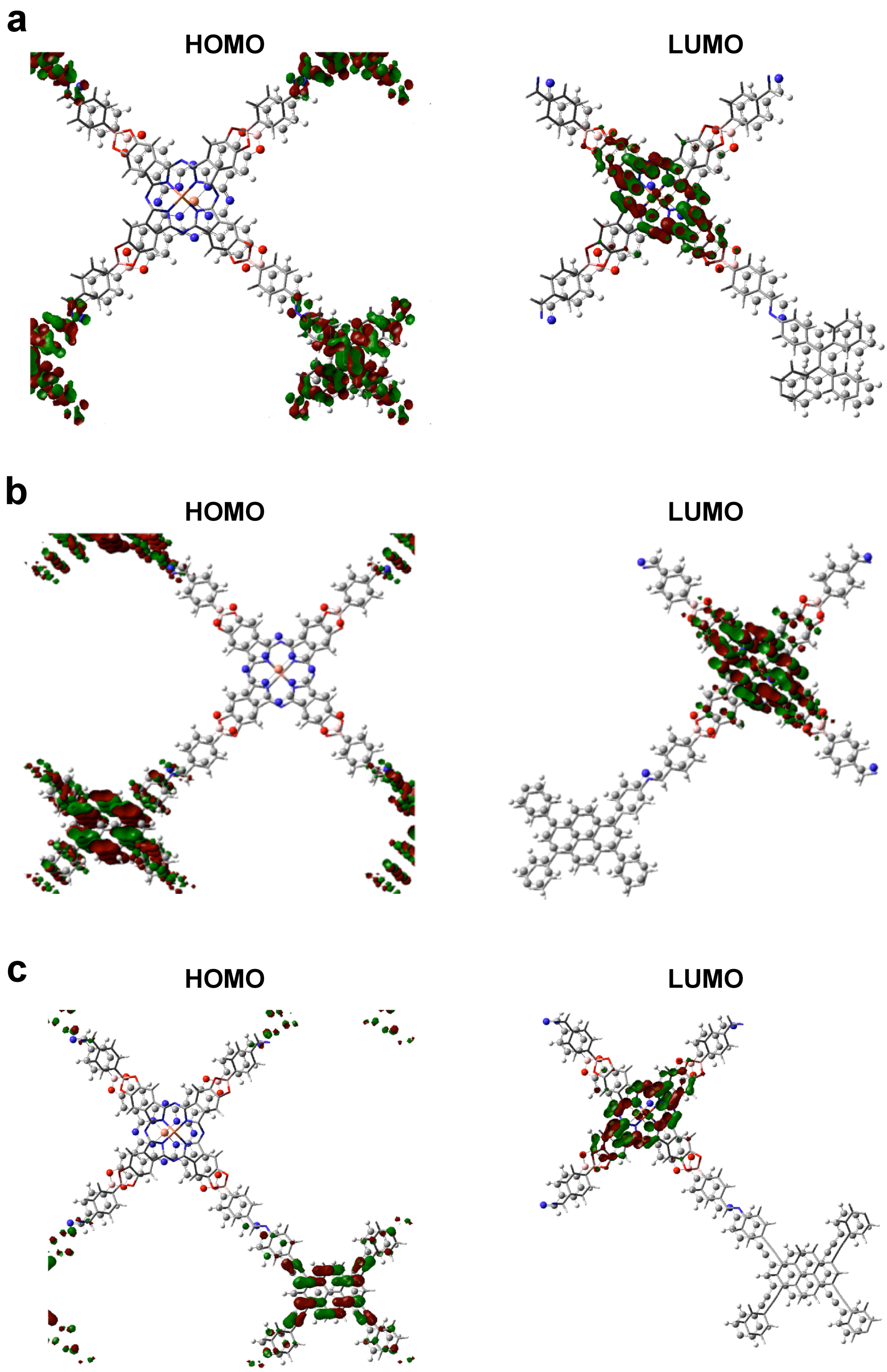

Figure S18. Frontier molecular orbitals of (a) the CuPc-FPBA-ETTA COF, (b) the CuP-FPBA-PyTTA COF and (c) the CuP-FPBA-TABPy COF. Bottom layer is depicted using ball-and-stick mode, top layer is shown using the tube representation.

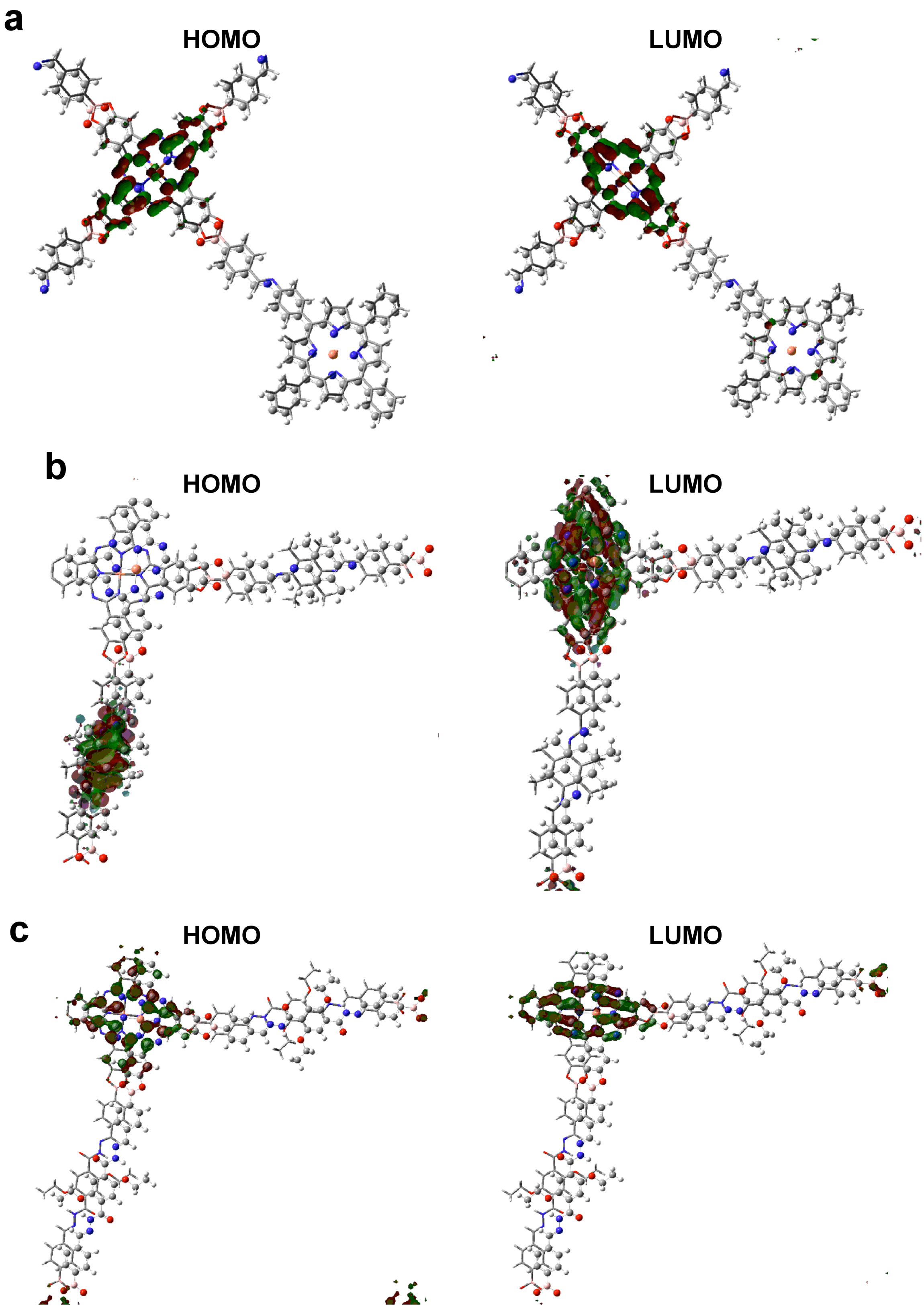

Figure S19. Frontier molecular orbitals of (a) the CuPc-FPBA-ZnP COF, (b) the CuP-FPBA-TMBDA COF and (c) the CuP-FPBA-DETHz COF. Bottom layer is depicted using ball-and-stick mode, top layer is shown using the tube representation.

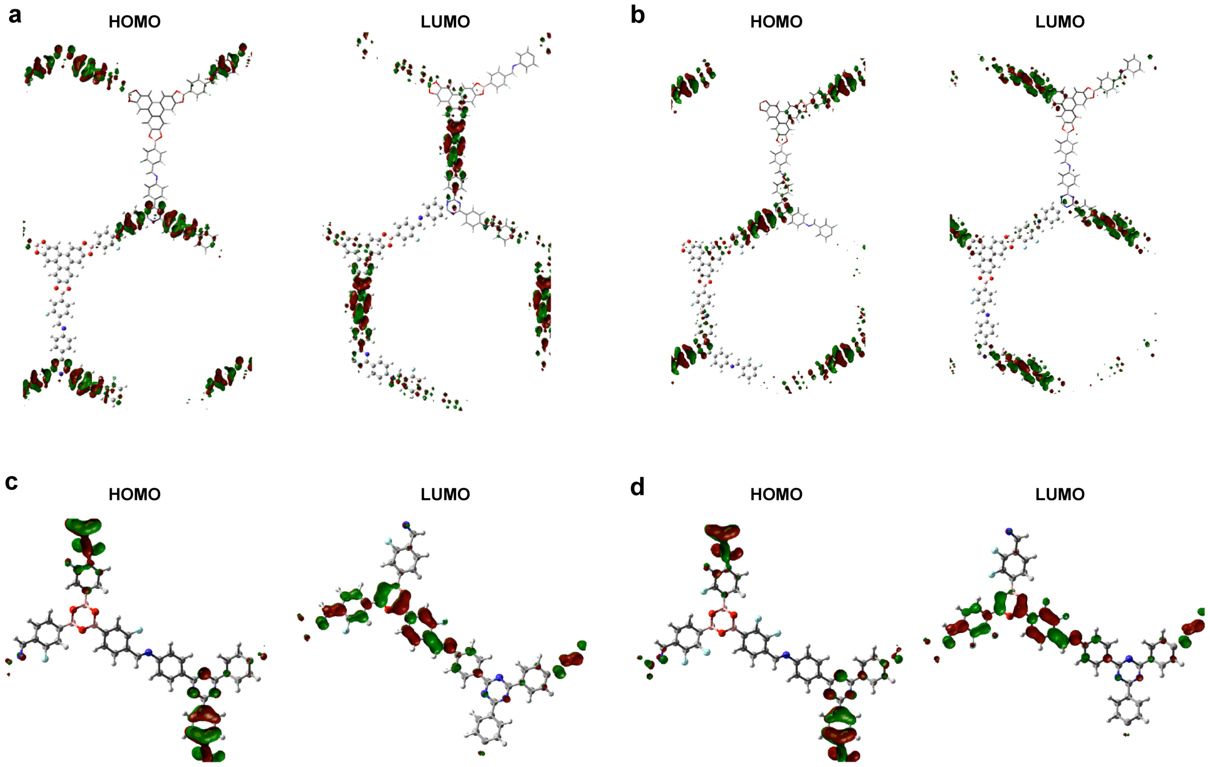

Figure S20. Frontier molecular orbitals of (a) the HHTP-FFPBA-TATTA COF, (b) the HHTP-DFFPBA-TATTA COF, (c) the TATTA-FFPBA COF and (d) the TATTA-DFFPBA COF. Bottom layer is depicted using ball-and-stick mode, top layer is shown using the tube representation.

Supplementary References

S1 Ding, X. *et al.* Synthesis and functions of two-dimensional metallophthalocyanine covalent organic frameworks with different metal ions. *Chem. Commun.* **48**, 8952-8954 (2012).

S2 Percec, V. *et al.* Self-assembly of dendronized triphenylenes into helical pyramidal columns and chiral spheres. *J. Am. Chem. Soc.* **131**, 7662-7677 (2009).

S3 Schreivogel, A., Maurer, J., Winter, R., Baro, A. & Laschat, S. Synthesis and electrochemical properties of tetrasubstituted tetraphenylethenes. *Eur. J. Org. Chem.* **2006**, 3395–3404 (2006).

S4 Chen, X. *et al.* Towards covalent organic frameworks with predesignable and aligned open docking sites. *Chem. Commun.* **50**, 6161-6163 (2014).

S5 Meng, W. *et al.* A Self-Assembled M_8L_6 Cubic Cage that Selectively Encapsulates Large Aromatic Guests. *Angew. Chem., Int. Ed.* **50**, 3479-3483 (2011).

S6 Wang, L. *et al.* A visible light excitable ''on–off'' and ''green–red'' fluorescent chemodosimeter for Ni^{2+}/Pb^{2+}. *New J. Chem.* **36**, 2176–2179 (2012).

S7 Gattuso, G. *et al.* Amino surface-functionalized tris(calix[4]arene) dendrons with rigid C_3-symmetric propeller cores. *Eur. J. Org. Chem.* **2011**, 5696–5703 (2011).

S8 Uribe-Romo, F. J., Doonan, C. J., Furukawa, H., Oisaki, K. & Yaghi, O. M. Crystalline covalent organic frameworks with hydrazone linkages. *J. Am. Chem. Soc.* **133**, 11478-11481 (2011).

S9 Aradi, B., Hourahine, B. & Frauenheim, T. $DFTB^+$, a sparse matrix-based implementation of the DFTB method. *J. Phys. Chem. A* **111**, 5678-5684 (2007).

S10 http://www.dftb.org.

S11 Cui, Q. Private communication.

S12 Addicoat, M., Vankova, N., Akter, I. S. & Heine, T. Extension of the universal force field to metal–organic frameworks. *J. Chem. Theory Comput.* **10**, 880-891 (2014).

S13 Gale, J. D. & Rohl, A. L. The general utility lattice program (GULP). *Mol. Simulat.* **29**, 291-341 (2003).

S14 Accelrys, Material Studio Release Notes, Release 4.4, Accelrys Software, San Diego (2008).
